# A calpain-6/YAP axis in sarcoma stem cells that drives the outgrowth of tumors and metastases

**DOI:** 10.1038/s41419-022-05244-3

**Published:** 2022-09-24

**Authors:** Joëlle Tchicaya-Bouanga, Yu-Jen Hung, Jean-Marc Schwartz, Diane Ji Yun Yoon, Emilie Chotard, Clarice Marty, Guillaume Anthony Odri, Gonzague de Pinieux, Martine Cohen-Solal, Dominique Modrowski

**Affiliations:** 1 Université Paris Cité, Inserm U1132 BIOSCAR, F-75010 Paris, France; 2 School of Biological Sciences, University of Manchester, Manchester, M13 9PT UK; 3 Institute of Biomedical Sciences, Academia Sinica, Taipei, Taiwan; 4 Service de chirurgie orthopédique et traumatologie, AP-HP, Hôpital Lariboisière, F-75010 Paris, France; 5 Service de rhumatologie, AP-HP, Hôpital Lariboisière, F-75010 Paris, France; 6 Department of Pathology, CHRU de Tours, Université de Tours, Tours, France

**Keywords:** Sarcoma, Cancer stem cells, Cell signalling

## Abstract

Sarcomas include cancer stem cells, but how these cells contribute to local and metastatic relapse is largely unknown. We previously showed the pro-tumor functions of calpain-6 in sarcoma stem cells. Here, we use an osteosarcoma cell model, osteosarcoma tissues and transcriptomic data from human tumors to study gene patterns associated with calpain-6 expression or suppression. Calpain-6 modulates the expression of Hippo pathway genes and stabilizes the hippo effector YAP. It also modulates the vesicular trafficking of β-catenin degradation complexes. Calpain-6 expression is associated with genes of the G2M phase of the cell cycle, supports G2M-related YAP activities and up-regulated genes controlling mitosis in sarcoma stem cells and tissues. In mouse models of bone sarcoma, most tumor cells expressed calpain-6 during the early steps of tumor out-growth. YAP inhibition prevented the neoformation of primary tumors and metastases but had no effect on already developed tumors. It could even accelerate lung metastasis associated with large bone tumors by affecting tumor-associated inflammation in the host tissues. Our results highlight a specific mechanism involving YAP transcriptional activity in cancer stem cells that is crucial during the early steps of tumor and metastasis outgrowth and that could be targeted to prevent sarcoma relapse.

## Introduction

Sarcomas are rare and heterogeneous tumors that appear in conjunctive tissues. These tumors are treated with aggressive chemotherapies and surgical resection, but local and metastatic relapses greatly reduces the patient survival [1, 2]. Despite the identification of many factors involved in metastatic properties of sarcoma cells the specific features of the disseminated cells that drive the progression of the disease progression and the formation of metastases remain unclear. Metastases and primary bone tumors have been shown to have common or different histology [3]. However, both are very heterogeneous tissues, which suggests that primary and metastatic tumors could follow the same maturation process. The main model for sarcoma development is the clonal one, however, cells with cancer stem cells (CSCs) features have been identified in osteo- and chondro- sarcomas and in Ewing sarcomas, so these tumors may also depend on a hierarchical organization [4–6]. At the opposite of carcinoma SCs shown to acquire an aggressive phenotype with increased migratory and dissemination capacities [7], whether and how sarcoma SCs could specifically contribute to metastasis formation is largely unknown. We previously showed that the atypical calpain, calpain-6, is expressed in sarcoma cells with CSC properties such as self-renewal, multipotency, chemoresistance and tumor initiation [8]. Calpain-6 could prevent apoptosis in uterine sarcoma and hypoxia-dependent senescence in osteosarcoma cells [8–10]. The functions of calpain-6 clearly link sarcoma SCs to tumor malignancy and possibly to the metastatic process.

The Hippo pathway is a conserved developmental program and a major regulator of organ size, tissue renewal and repair [11]. The Hippo effectors YAP and TAZ in complex with transcription factors such as TEAD modulate the expression of genes that are lineage determinants, self-renewal factors, and apoptosis-related genes and thus have key functions to control embryonic SC fate and survival [12, 13]. However, YAP can either maintain a stem/progenitor state or stimulate differentiation, depending on the context. The Hippo pathway has tumor suppressor functions, and the activities of YAP were found associated with tumor progression, metastasis, chemoresistance and cancer stemness [14]. The expression of genes of the Hippo cascade were found altered in sarcomas [15]. Tissue microarrays showed high levels of YAP correlated with staging in human osteosarcomas [16, 17]. YAP target genes were also found up-regulated in bone tumors [18]. Finally, inhibition of the Hippo pathway was reported to promote stemness in cancer cells [19]. Together these data raise the question of the possible involvement of YAP in specific functions of sarcoma SCs.

Here, we report that calpain-6 and YAP expression are correlated in osteosarcoma cells and tissues. Calpain-6 stabilized YAP by regulating the Hippo pathway and the recycling of the β-catenin-degradation complex via intracellular vesicles. The calpain-6/YAP axis was associated with the G2M phase of the cell cycle and regulated genes that control mitosis. YAP inhibition increased the rate of mitotic catastrophes in vitro and suppressed the neo-formation of bone tumors and lung metastases in mice but could also promote tumor-associated inflammation in the host tissues and increase tumor cell proliferation.

## Results

### Calpain-6 expression is associated with an altered hippo pathway in CSCs

**Fig. 1:**
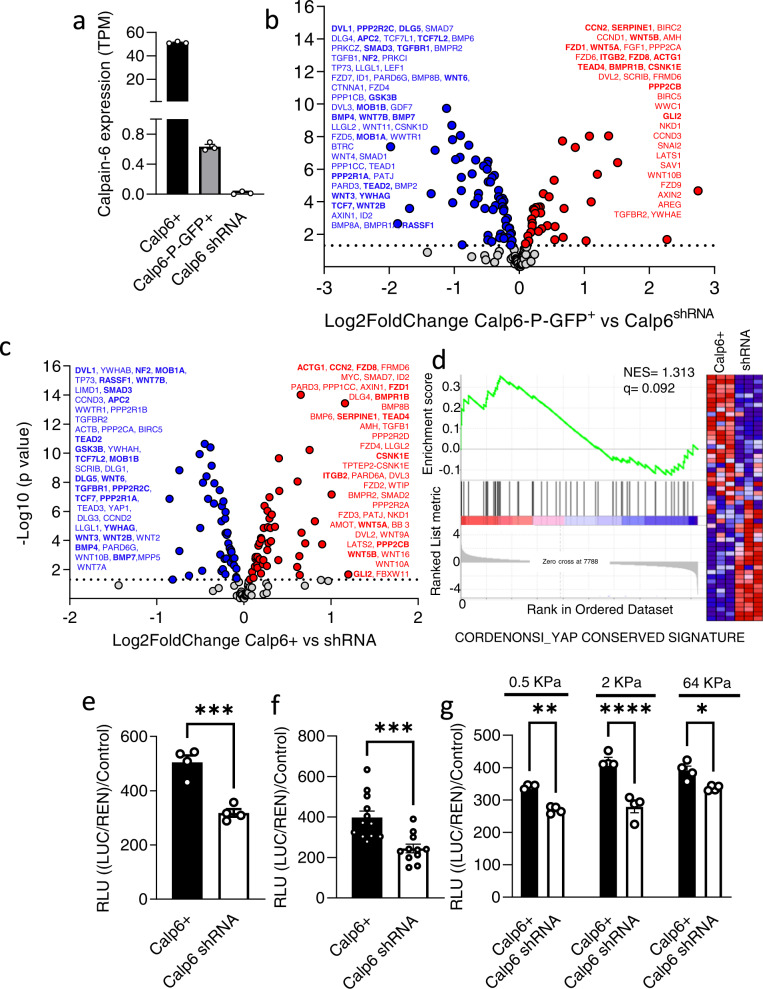
Calpain-6 overexpression is associated with high YAP/TEAD activity in osteosarcoma cells. **a** Calpain-6 mRNA expression in calpain-6–overexpressing cells (Calp6 + ), calpain-6-expressing cells (Calp6-P-GFP + ; GFP + ) and calpain-6-knockdown cells (Calp6 shRNA). TPM: Transcripts per kilobase million. **b**, **c** Volcano plots from RNAseq analysis showing Hippo pathway genes differentially expressed in Calp6-P-GFP + (**b**) or Calp6 + (**c**) versus Calp6 shRNA cells. Red and blue dots, *p* < 0.05, grey dots, *p* > 0.05. Dotted line indicates the threshold -Log2(*p* = 0.05). The lists recapitulate the differentially expressed genes ranked from higher to lower -Log2(*p* value). Common genes between Calp6+ and Calp6-P-GFP + cells are in bold. **d**, Gene set enrichment analysis of a YAP target genes signature comparing Calp6+ to Calp6 shRNA cells. NES is normalized enrichment score. **e**–**g**, YAP/TEAD-dep**e**ndent transcriptional activity in Calp6+ and Calp6 shRNA 143B (**e**, **g**) or U2OS (**f**) cells. Cells were plated on regular plastic (**e**,**f**) or on collagen-coated silicon (**g**) and transfected with GT11c (TEAD) and Renilla plasmids. Stiffness of the collagen-coated matrix in kPa is indicated. Results are mean ± SEM relative luminescence unit (RLU). Renilla activity was a transfection control. * <0.05, **p < 0.01, ***p < 0.001, ****p < 0.0001.

To determine active and targetable mechanisms that contribute to specific pro-tumor functions of sarcoma stem cells, we used the CSC biomarker calpain-6. Because we previously showed that calpain-6 inhibition suppressed the tumor-initiating properties of osteosarcoma cells, we compared transcriptomes of cells that express or overexpress calpain-6 with those of calpain-6 knockdown cells. Only part of the cell population expresses calpain-6 in osteosarcoma cell lines [8]. Therefore, we used the 143B human osteosarcoma cell line that was modified to express GFP under control of the CAPN6 regulatory sequence to sort and enrich a CSC population expressing basal levels of calpain-6 (Calp6-P-GFP + cells) (Fig. 1a, Supplementary Fig. 1). We compared these cells to a homogeneous population with calpain-6 expression stably inhibited by a specific shRNA (Calp6^shRNA^ cells) (Fig. 1a, Supplementary Fig. 1). We also created a homogeneous population of 143B cells that overexpressed calpain-6 by using a lentiviral vector (Calp6 + cells) (Fig. 1a, Supplementary Fig. 1).

After checking that the RNAseq analysis passed quality controls, the lists of differentially expressed genes were ordered on the basis of log2fold-changes. Consistent with previous findings for calpain-6, biological terms associated with differentially expressed genes included those that regulate the organization of microtubules, RNA splicing and autophagy (Supplementary Fig. 2a, d). This finding validated our cellular models to further study calpain-6-expressing cells. KEGG analyses revealed an undiscovered association between calpain-6 and genes of the Hippo pathway (Supplementary Fig. 2b, e). Among the 153 genes of the Hippo signaling signature, 94 were significantly differentially expressed in Calp6-P-GFP + cells and 92 in Calp6+ cells as compared with Calp6^shRNA^ cells (Fig. 1b, c). The genes NF2, MOB1A, TEAD2, Wnt and BMP factors were downregulated, and TEAD4, CCN2 and Serpin1 were up-regulated in calpain-6 expressing and overexpressing cells versus Calp6^shRNA^ cells (Fig. 1b, c). Moreover, gene set enrichment analysis (GSEA) showed a significant enrichment for CORDENONSI_YAP_CONSERVED_ SIGNATURE among the up-regulated genes in Calp6+ cells (Fig. 1d). RT-PCR analyses confirmed that YAP/TEAD targets such as CTGF, Cyr61 and ANKRD1 were up-regulated in Calp6 + 143B and U2OS cells (Supplementary Fig. 3a, b). Another set of shRNA that inhibited calpain-6 expression had similar effects on YAP target genes expression confirming the specific effect of calpain-6 suppression on YAP activity (Supplementary Fig. 3c, d). In contrast, calpain-6 overexpression was associated with higher TEAD transcriptional activity (GT11c) in different osteosarcoma cell lines (Fig. 1e, f). YAP is a mechano-sensor whose activity is regulated by the physical properties of the environment. To rule out a possible overstimulation due to the high stiffness of the regular culture plates and determine the regulation of YAP activity by calpain-6 in different tissues such as brain, lungs and the osteoid part of the bone, we measured GT11c activity in cells cultured on collagen-coated silicone with different stiffness. Calp6+ cells had higher TEAD activity than Calp6^shRNA^ cells on 0.5, 2 and 64 KPa matrix (Fig. 1g).

### Calpain-6 stabilized YAP protein

**Fig. 2:**
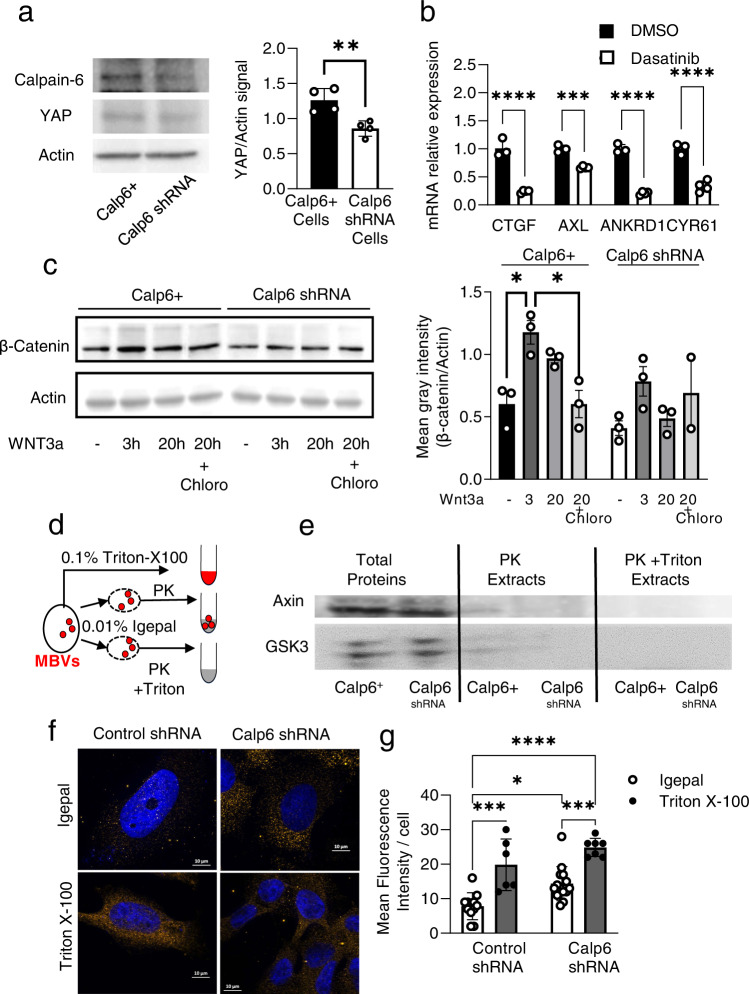
Calpain-6 stabilizes YAP protein. **a** Proteins were extracted from Calp6+ and Calp6 shRNA cells and used for Calpain-6 and YAP immunoblot assays. Actin was a loading control. Panel on the right shows the quantification of 4 different Western blots. **b** Effect of Hippo pathway activation with Dasatinib on the expression of YAP/TEAD targets as indicated in calpain-6 overexpressing 143B cells. Control cells were treated with the eluent DMSO. Results are expressed as mean fold changes (ΔΔCt) ± SEM. **c** 143B cells were treated with medium conditioned on WNT3a-producing cells or control cells in the presence of DMSO or chloroquine. Protein extracts were prepared 3 or 20 h after Wnt stimulation for β-catenin immunoblot assay. Results are mean ± SEM relative grey intensity of β-catenin to actin from 3 independent blots. **d** Proteinase K protection assay. Cells were lyzed with triton X-100 to obtain whole protein extracts or with a soft detergent to prepare intact multivesicular bodies (MBVs). Proteinase K (PK) was added to digest proteins that were not protected into MBVs. The addition of Triton with PK served to control MBV-dependent protection. **e**, Immunoblots for Axin and GSK3 in the 3 types of protein extracts. **f**, GSK3 immunofluorescence in Calp6-P-GFP + 143B cells expressing control or Calp6 shRNA. The cells were permeabilized with Igepal or Triton X-100 before incubation with anti-GSK3 antibodies. Dapi served to stain the nuclei. **g**, Quantification of the mean fluorescence in GFP + cells. *, *** and **** indicate significant difference, *p* < 0.05, *p* < 0,001 and *p* < 0,0001, respectively.

We then investigated mechanisms that could lead to the increased YAP activity in Calp6-P-GFP + cells. The expression of YAP mRNA was not significantly different in Calp6+ and Calp6^shRNA^ cells (Supplementary Fig. 3e). However, western blot analysis showed higher level of YAP protein in Calp6+ versus Calp6 ^shRNA^ cells (Fig. 2a, supplementary data). In the same way, the YAP paralogue TAZ, with redundant activities, was upregulated in Calp6+ cells (Supplementary Fig. 3f, supplementary data). The RNA-seq analysis suggested that alterations of the Hippo pathway contributed to YAP stabilization. However, Dasatinib, a Hippo pathway stimulator [20] was able to inhibit the expression of YAP target genes CTGF, AXL, ANKRD1 and CYR61 in Calp6+ and Calp6-P-GFP + cells (Fig. 2b, Supplementary Fig. 4a). Verteporfin, a direct inhibitor of YAP and TAZ activity, downstream of the phosphorylation cascade, also reduced the expression of CTGF, AXL and CYR61 in Calp6+ cells but this treatment prevented the extraction of good quality mRNA from Calp6-P-GFP + cells suggesting that it induced cell death. It also suggested that other mechanisms independent from the Hippo pathway may contribute to an abnormal activity of YAP/TAZ in CSCs. The degradation of YAP depends on it being addressed to the proteasome by the β-catenin degradation complex GSK3/AXIN/APC, but basal β-catenin level was increased in Calp6+ versus Calp6 ^shRNA^ cells (Fig. 2c, Supplementary data). In addition, stimulation with Wnt3a increased β-catenin level in Calp6 ^shRNA^ and Calp6+ cells at 3 hr after its addition in the culture medium (Fig. 2c, Supplementary data). At 20 hr after Wnt stimulation, β-catenin returned to its basal level only in Calp6^shRNA^ cells and remained higher in Calp6+ cells (Fig. 2c, Supplementary data). Sequestration of the β-catenin degradation complex in multivesicular bodies (MVBs) of the endosomal compartment can block β-catenin degradation and promote Wnt signaling [21]. Gargini et al. demonstrated that this sequestration can be also responsible for YAP stabilization [22]. Inhibiting the receptor recycling process with chloroquine in 143B Calp6+ cells restored the reduction of β-catenin level 20 hr after Wnt stimulation (Fig. 2c, Supplementary data) which suggests that calpain-6 could affect the recycling of the β-catenin degradation complex. We then performed a proteinase K protection assay that showed GSK3 and Axin still trapped in MVBs of Calp6+ but not of Calp6 ^shRNA^ cells at 20 hr after the addition of Wnt3a to the medium (Fig. 2d, e, Supplementary data). In the same way, we performed GSK3 immunofluorescence in Calp6-P-GFP cells expressing control or Calp6 shRNA treated for 8 h with Wnt3a. Higher levels of GSK3 were detected in Calp6 shRNA compared to control shRNA Calp6-P-GFP + cells after soft permeabilization with Igepal (Fig. 2f, g). Triton increased the GSK3 signal at the same levels in the different types of cells, showing that, in control Calp6-P-GFP + cells, a greater amount of GSK3 was protected in vesicles unless we performed a strong permeabilization with Triton (Fig. 2f, g). Together our findings indicate that calpain-6 controlled the fate of the β-catenin degradation complex and thus promotes YAP accumulation in CSCs.

### Calpain-6-dependent promotion of YAP activity was associated with the G2M cell cycle phase

**Fig. 3:**
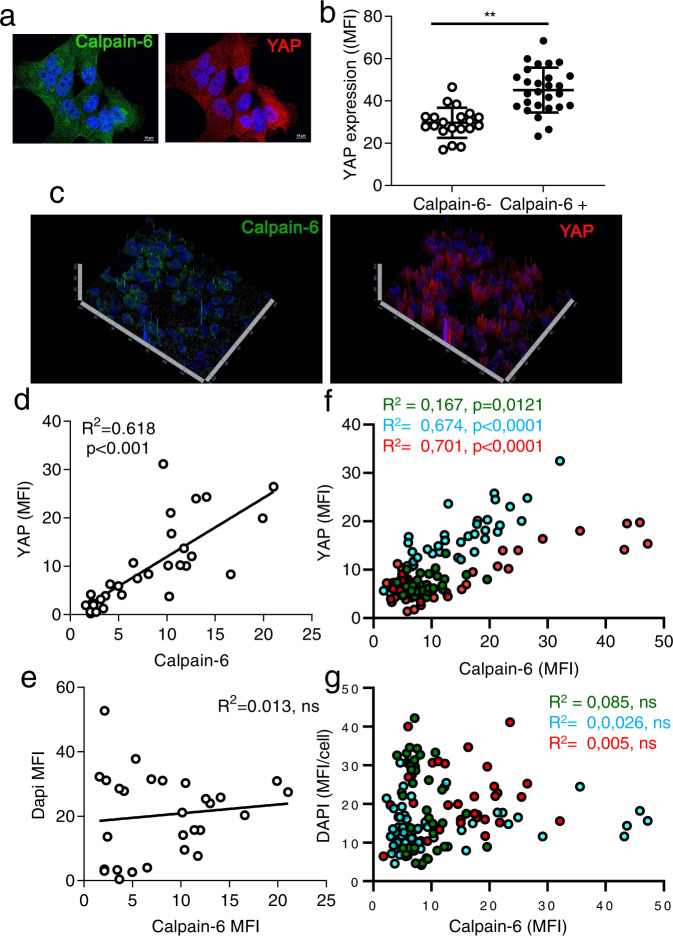
YAP accumulates in calpain-6–expressing cells and tumors. **a** Representative immunofluorescence of calpain-6 (green) and YAP (red) in cultured 143B cells. DAPI (blue) staining indicates nuclei. Green arrow indicates calpain-6 and corresponding YAP labeling in the same cell. **b** Mean ± SEM fluorescence intensity (MFI) of YAP labeling in cells without calpain-6 (Calpain-6-) and in calpain-6–expressing cells (Calpain-6 + ). **c** Representative 3D view of calpain-6 (green) and YAP (red) labeling in a section of 143B cell-derived bone tumor in mouse. **d**, **e**, Correlations between Calpain-6 and YAP (**d**) or DAPI (**e**) staining in cells of the mouse tumor section. **f**, **g**, Correlations between Calpain-6 and YAP (**f**) or DAPI (**g**) staining in cells of tumor sections from paraffin-embedded lung metastatic tissue from osteosarcoma patients. The colors of the different dots distinguish cells of tissue sections from 3 different patients.

**Fig. 4:**
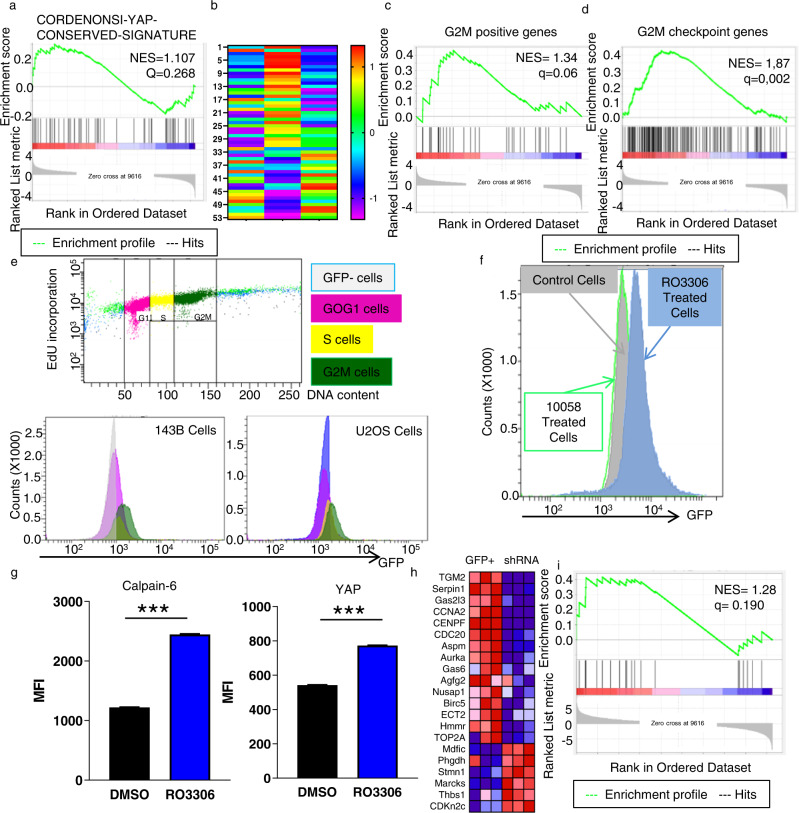
Calpain-6–dependent promotion of YAP protein is associated with the G2M phase. **a** Analysis of the CORDENONSI_YAP_CONSERVED_ SIGNATURE in Calp6-P-GFP + vs Calp6 shRNA cells. **b** Heat map showing the CORDENONSI_YAP_CONSERVED_ SIGNATURE in calpain-6–overexpressing (Calp6 + ), Calp6-P-GFP + (GFP + ) and Calp6 shRNA–expressing cells (Calp6 shRNA). **c**, **d**, Gene set enrichment analysis of G2M-positive and G2-checkpoint gene signatures comparing Calp6-P-GFP + and Calp6 shRNA cells. NES is normalized enrichment score. **e** Flow cytometry of expression of GFP in Calp6-P-GFP cells during the cell cycle. Analysis of DNA content and Edu incorporation to select cells in the different phases of the cycle and GFP fluorescence intensity in different sorted cell populations. Color code for dots and histograms: Pink=G1, yellow = S, Green=G2M. Grey or blue histograms are negative controls. **f** GFP fluorescence in Calp6-P-GFP cells that were blocked in G1 (10058 treated cells, green histograms), in G2 (R03306 treated cells, blue histograms) and in control cells (grey histograms). **g** Calpain-6 and YAP labeling in control or G2M-blocked (RO3306) cells. Results are mean ± SEM fluorescence intensity (MFI). 45000 cells were analyzed. ***p < 0.001. **h** Heat map of the differential expression of YAP-dependent mitotic genes in Calp6-P-GFP + and Calp6 shRNA cells. **i**, Mitotic gene enrichment analysis comparing Calp6-P-GFP + to Calp6 shRNA cells. **h**, **i**, From t**h**e left, the list of the genes, heat map of gene expression and enrichment curve.

The accumulation of YAP protein did not only result from calpain-6 overexpression because immunofluorescence showed that level of YAP was higher in cells expressing basal levels of calpain-6 than cells without calpain-6 (Fig. 3a, b). Moreover, calpain-6 and YAP levels were correlated in a 143B cells-derived bone tumor in mouse and in osteosarcoma lung metastases in patients (Fig. 3c, d, f and Supplementary Fig. 5). Of note, these correlations were not a bias due to heterogeneity of tissues labeling because DAPI staining was not related to Calpain-6 staining (Fig. 3e, g). GSEA showed no statistically significant enrichment of CORDENONSI_YAP_CONSERVED_ SIGNATURE in cells expressing basal levels of calpain-6 (Calp6-P-GFP + cells) as compared with Calp6 ^shRNA^ (Fig. 4a). In contrast, the pattern of expression of YAP target genes differed in Calp6-P-GFP + and Calp6+ cells, which indicates that YAP stabilization in calpain-6-expressing and -overexpressing cells did not result in the same transcriptional regulation (Fig. 4b). It was previously reported that calpain-6 overexpression blocked cytokinesis suggesting that calpain-6 expression should be fine-tuned over the cell cycle to ensure normal functions [23]. Reactome Pathway Analysis highlighted a differential expression of genes involved in the Mitotic G2/M phase, G2M transition and M phase in Calp6-P-GFP + cells (Supplementary Fig. 2c, f). GSEA further showed that basal calpain-6 expression but not overexpression in 143B cells was associated with a gene signature related to positive regulation of the G2M phase (Fig. 4c). Calpain-6 was also associated with the expression of genes of the G2M checkpoints (Fig. 4d). Flow cytometry analyses of Calp6-P-GFP + cells showed that the highest GFP expression was observed in cells in G2M (Fig. 4e). Moreover, GFP expression was increased in calp6-P-GFP + cells that were blocked in G2M with a CDK1 inhibitor, RO3306, but not in cells blocked in G1 with a Myc inhibitor (Fig. 4f). Thus calpain-6 was mainly produced at the G2M phase of the cell cycle. Consistently, YAP protein level was enhanced along with calpain-6 in G2M-blocked cells (Fig. 4g). Hence, the calpain-6-dependent increase in YAP protein level in CSCs seemed mainly associated with the G2M phase of the cell cycle.

### YAP controls the expression of mitotic genes in calpain-6 expressing cells

**Fig. 5:**
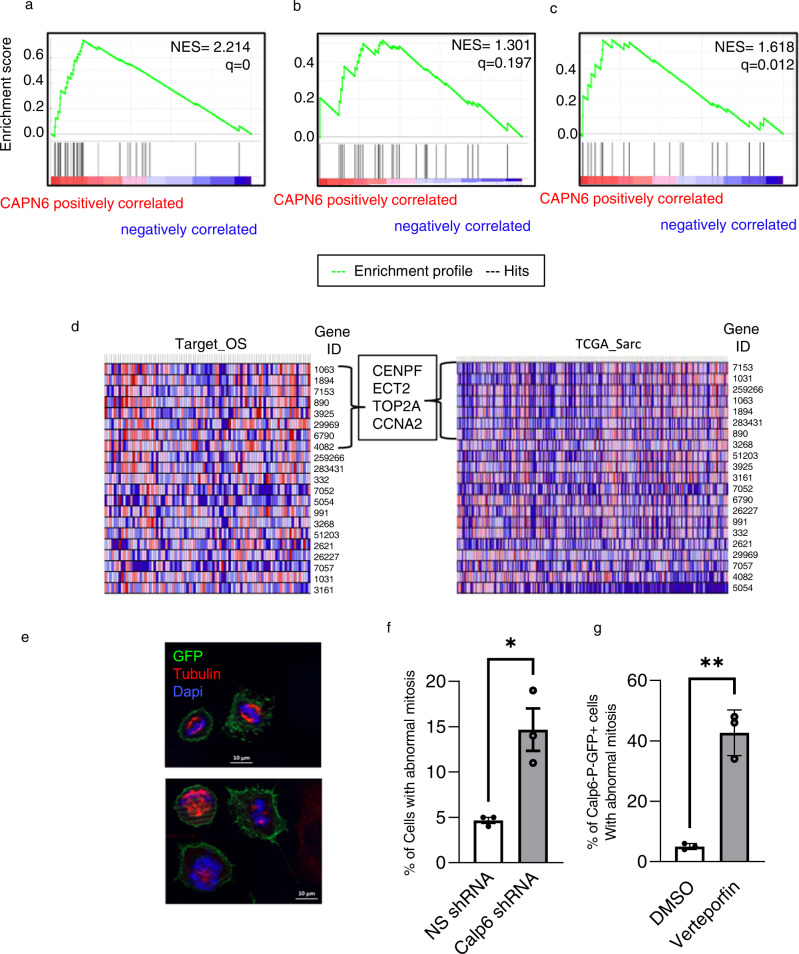
Calpain-6 expression is associated with upregulation of G2M and mitotic genes in sarcomas. Calpain-6–dependent gene set enrichment in sarcoma tissues. **a**, **b** Enrichment of G2M-positive genes in soft-tissue sarcoma (TCGA-SARC) (**a**) and in osteosarcomas (Target-OS) (**b**). **c** Mitotic gene set enrichment in soft-tissue sarcomas. **d** Heat map of variation in expression of mitotic genes (Entry gene ID as indicated) according to calpain-6 expression in osteosarcomas and soft-tissue sarcomas. **e** Mitosis was observed in Calp6-P-GFP + cells. DAPI staining (blue) shows the morphology of nuclei and immunofluorescence labeling of α-tubulin (red) shows mitotic spindle. Upper panel shows normal mitosis figures in control cells; lower panels show abnormal mitosis figures representative of mitotic catastrophe in verteporfin-treated or calpain-6 shRNA-expressing cells. **f, g** The cells were blocked in G2 overnight. Fresh medium was then added to induce the entry into mitosis. The percentage of cells with abnormal mitosis was counted comparing control and Calp6 shRNA-expressing (**f**) and DMSO vs verteporfin-treated 143B Calp6-P-GFP + cells (**g**). Results are means ± SEM of the percentage of catastrophe mitosis in 3 different cultures. *p < 0.05, **p < 0.01.

The results presented above led us to investigate whether YAP or calpain-6 inhibition could affect G2M-related functions in CSCs. Recently, YAP was found to regulate mitotic genes by promoting interactions between the Myb-MuvB complex and distal enhancers [24]. In contrast to CORDENONSI_YAP _CONSERVED_SIGNATURE, YAP-dependent mitotic genes were significantly enriched in Calp6-P-GFP + versus Calp6 ^shRNA^ cells (Fig. 4h). We then used transcriptomic data from a collection of 88 osteosarcomas (Target OS) and 275 soft-tissue sarcomas (TGCA-Sarc) to further characterize the possible involvement of YAP in CSC populations. The tumors were ranked according to calpain-6 expression (Supplementary Fig. 6a, b). We did not find a positive association between levels of calpain-6 and the CORDENONSI_YAP _CONSERVED_SIGNATURE, but rather a correlation with levels of G2M-associated genes in the different sarcomas types (Fig. 5a, b; Supplementary Fig. 6c, d). Of note, calpain-6 expression in cells and in sarcoma tissues was associated with that of genes involved in DNA repair (Supplementary Fig. 7a–c). We also found a strong enrichment in a YAP-dependent mitotic gene signature in calpain-6 expressing soft-tissues sarcomas (Fig. 5c). Although, GSEA did not show a clear enrichment with this signature in osteosarcomas, CENPF, ECT2, TOP2A, and CCNA2 were up-regulated in calpain-6 expressing bone and soft-tissue sarcomas (Fig. 5d). Together these results highlighted the key functions of the calpain-6/YAP axis during the G2M phase in sarcoma stem cells. Because the mitotic genes code for factors that control mitosis, we hypothesized that the calpain-6/YAP axis plays a crucial role during tumor or metastasis outgrowth by protecting the cells against mitosis-associated cell death. In support of this, we observed that the expression of genes coding the anti-apoptotic factors Survivin, BCL2 and MCL1 was down-regulated in Calp6 ^shRNA^ cells, whereas pro-apototic factors such as Bad and caspase-8 were up-regulated in these cells as compared with Calp6-P-GFP + cells (Supplementary Fig. 7d). Moreover, calpain-6 suppression with shRNA or YAP inhibition with verteporfin increased the rate of mitotic catastrophe in Calp6-P-GFP + 143B dividing cells (Fig. 5e, f, g, Supplementary Fig. 7e).

### Calpain-6/YAP axis is required for tumor and metastasis out-growth

**Fig. 6:**
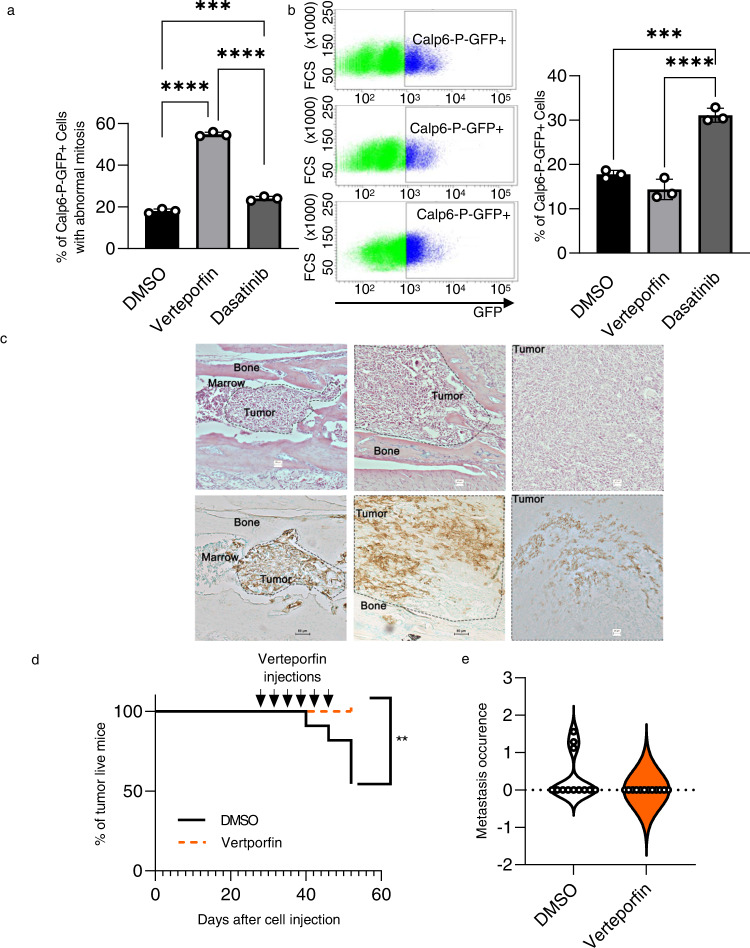
Calpain-6/YAP axis is involved in bone tumor out-growth. **a** The effects of YAP inhibitors Dasatinib and Verteporfin vs the eluent (DMSO) on mitosis was observed in Calp6-P-GFP + K7M2 cells. The percentage of cells with abnormal mitosis was counted. Results are means ± SEM of the percentage of catastrophe mitosis in 3 different cultures. ***p < 0.001, ****p < 0.0001. **b** The effect of Dasatinib and Verteporfin on the % of K7M2 Calp6-P-GFP + cells. The cells were treated for 3 days with a YAP inhibitor or the eluent (DMSO) and the GFP + cells were counted by cytometry. **c** Tumor evolution in bone at different times after the implantation of Calp6-P-GFP K7M2 cells. Upper panels show H&E staining of bone sections 7 and 14 days after cell implantation and large/mature bone tumor. Lower panels show GFP immunohistochemistry (brown staining) in corresponding adjacent sections. Dotted lines indicate the extent of the tumor. **d** Mice with slowly developing tumors were treated with DMSO or verteporfin as indicated (arrows). The survival curves indicate the percentage of living mice. *n* = 12 DMSO-treated mice, *n* = 11 verteporfin-treated mice. **p < 0.01. **e** Quantification of lung metastases in these mice.

We previously reported that calpain-6 inhibition in K7M2 osteosarcoma cells suppressed their ability to form bone tumors and develop metastases in mice [8]. Verteporfin treatment promoted the rate of mitotic catastrophes in Calp6-P-GFP + K7M2 cells and tended to reduce this cell population after a few days of culture (Fig. 6a, b). At the opposite, Dasatinib had a very modest effect on mitotic catastrophe and resulted in a strong increase in the Calp6-P-GFP + K7M2 cell population (Fig. 6a, b). We therefore used verteporfin to access the in vivo effect of YAP inhibition to target CSCs in a model of bone tumor that consisted of intratibial implantation of Calp6-P-GFP K7M2 cells. In this syngeneic model, the tumors developed at a different rate: quickly during the 4 first weeks or only 5–7 weeks after cell implantation. Immunohistochemistry of GFP at different times after cell implantation in bone revealed that most of the tumor cells contributing to tumor initiation in the marrow were calpain-6 expressing cells, whereas this specific cell population decreased during tumor progression (Fig. 6c). In large bone tumors (≥520 mm^3^) GFP expression was much more variable and could persist only in some cell clusters (Fig. 6c). We treated K7M2 cells-implanted mice with verteporfin to assess the effects of YAP inhibition on tumors at different stages of development (Supplementary Fig. 8a, b). When the treatment was administrated at early times, that is, 7 days after cell implantation, or 30 days after cell implantation in mice that still had no detectable bone tumor, verteporfin prevented tumor growth (Fig. 6d, Supplementary Fig. 8d–f).

**Fig. 7:**
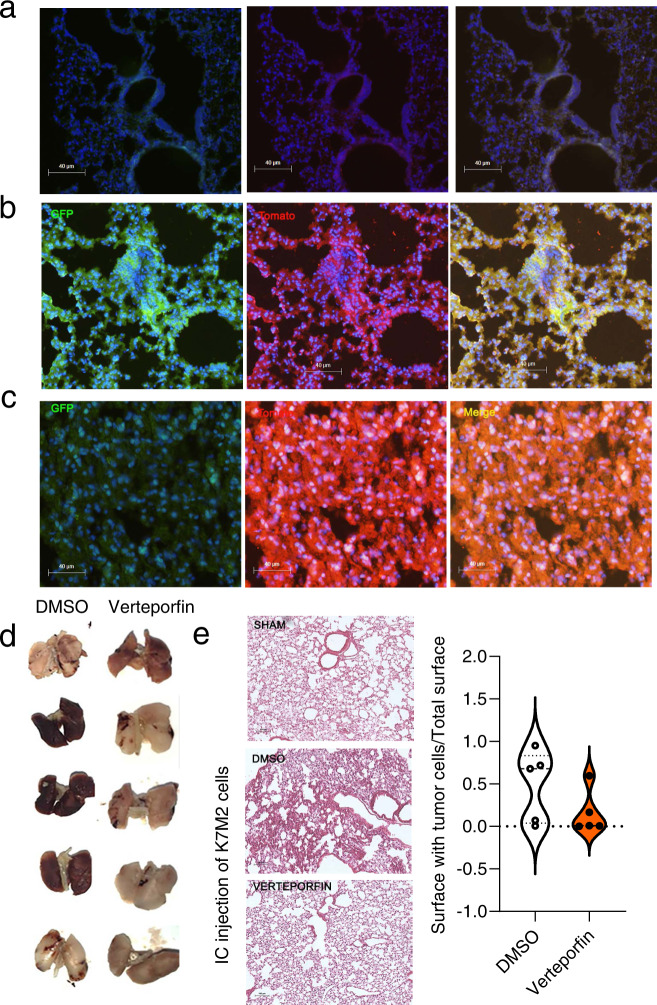
Calpain-6/YAP axis is involved in metastasis out-growth. **a–c** Detection of tumor cells in lungs after intracardiac injection of PBS (**a**) or K7M2 cells (**b**, **c**). Lungs of injected mice were collected and paraffin-imbedded 11 days or 5 weeks after cell injection. Tomato (red) and GFP (green) immunofluorescence shows tumor and Calp6-P-GFP + cells, respectively. **d** Photos of lungs 34 days after intracardiac (IC) injection. Mice were treated with DMSO or verteporfin as indicated. **e** Representative photos of lung sections from DMSO- vs verteporfin-treated mice 5 weeks after IC injection of K7M2 cells (H&E staining) and quantification of the surfaces of lungs with tumor cells.

To determine whether verteporfin treatment affected the metastasis process, we examined ex vivo cultures of lung cells from mice with slowly developing tumors (treatment at 28 days after cell implantation). At the end of the experiment, half of the lungs were dissociated and cultured in the presence of G418 to select tumor cells. Clones of selected cells were isolated from the lungs of 3 to 11 control mice (DMSO group), but no cells grew from the lungs of verteporfin-treated mice. As expected H&E staining of the other part of the lungs revealed metastatic nodules only in the 3 DMSO-treated mice (Fig. 6e). In contrast, only 2 to 5 mice that were treated with verteporfin 7 days after cell implantation had lung metastases, but 4 to 5 control mice had lung nodules (Supplementary Fig. 8f). Together these results suggest that YAP inhibition also prevented disseminated disease. To further document this possible role of the calpain-6/YAP axis during metastasis outgrowth, we intracardially (IC) injected calp6-P-GFP K7M2^EF1Tomato^ cells in mice (Supplementary Fig. 7c). Eleven days after IC injection, clusters of Tomato + /Calp6-P-GFP + cells were detected within mouse lungs, whereas, 5–6 weeks after the IC injection the lungs of control mice were invaded with tumors cells that were mostly Calp6-P-GFP- (Fig. 7a–c). Verteporfin treatment started 11 days after IC cell injection prevented the expansion of tumor cells within the lungs (Fig. 7d, e). Thus, YAP inhibition could block early stages of the tumorigenic process that involved mainly calpain-6 expressing cells in bone and lungs.

### The effects of verteporfin on tumors depends on the inflammatory context

**Fig. 8:**
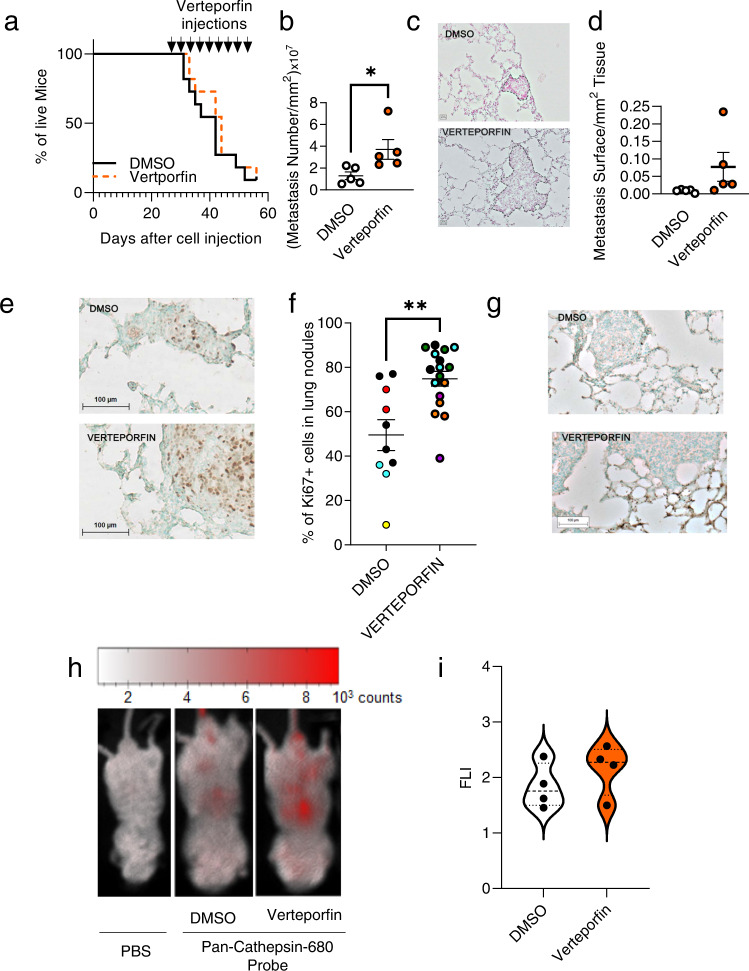
The effects of verteporfin on developed tumors is associated with inflammation. **a** Mice with bone tumors were treated with DMSO or verteporfin as indicated (arrows). Survival curves indicating the proportion of living mice. *n* = 11 DMSO-treated mice, *n* = 11 Verteporfin-treated mice. **b–d** Quantification of lung metastases in these mice. **b** Results are mean ± SEM number of lung nodules per mm^2^ of analyzed tissue. **c** Representative photos of lung tumor nodules in DMSO- (upper panel) and verteporfin- (lower panel) treated mice. **d** Results are mean ± SEM surface of lung nodules per mm^2^ of analyzed tissue. **e** Representative photos of Ki67 immunostaining in lung metastases of DMSO- and verteporfin-treated mice. **f** Percentage of Ki67-positive cells in lung metastases of DMSO- vs verteporfin-treated mice. Results are mean ± SEM. **p < 0.01. Colored dots discriminate the 3 different mice analyzed. **g** Representative photos of NF-KB (p65) labelling in cells around the metastases in lungs of DMSO- vs verteporfin-treated mice. **h** Detection of cathepsin activity in mice with fast-growing bone tumors treated with DMSO or verteporfin as indicated. At 24 h after NIR imaging, the mice were injected with PBS (sham injected) or a pan-cathepsin probe. **i** Quantif**i**cation of the NIR signal in lungs of DMSO- vs verteporfin-treated mice.

In mice with tumors before the treatment was started, verteporfin did not stop bone tumor growth (Fig. 8a). Moreover, tumor-bearing mice that were treated with verteporfin tended to have more and larger lung metastases than the control mice (Fig. 8b–d). Lung metastases from these verteporfin-treated mice contained a higher number of Ki67+ cells which suggests that verteporfin rapidly induced cell proliferation (Fig. 8e, f). However, in vitro, verteporfin had no effect on the proliferation of Calp6-P-GFP + or GFP- K7M2 cells (Supplementary Fig. 9a). This finding suggests that the proliferation of tumor cells could be indirectly induced by this compound in vivo. Because YAP inhibition prevents the resolution of inflammation in lungs and joints [25–27], we compared inflammation in lungs of mice with fast-growing tumors that were treated with DMSO or verteporfin. H&E staining and immunolabeling of nuclear p65 NF-kB showed that metastases from verteporfin-treated mice were surrounded with numerous inflammatory cells as compared with control mice (Fig. 8g; Supplementary Fig. 9b). Using a fluorescent probe to assess pan-cathepsin activity, we confirmed that YAP inhibition could increase lung inflammation in the mice with fast-growing tumors (Fig. 8h, i).

## Discussion

We reveal a novel axis involving calpain-6 and YAP that is activated in CSCs during the early steps of primary and metastatic tumor outgrowth. Tumor cell clones do not evolve independently but are linked by hierarchical relationships and derive or “differentiate” from each other [28, 29]. Recently, single-cell RNAseq analyses allowed for the identification of diverse osteosarcoma cell populations and showed that some specific tumor cells are at the center of a differentiation process [30, 31]. Together, these findings support a concept of tumor stem/precursor-like cells. Although CSCs can be very small populations at diagnosis, they are believed to contribute to chemoresistance and disease relapses [32]. Therefore, we need to identify specific mechanisms that could be targeted in CSCs to prevent these events that are responsible for patient mortality. Our results strengthen previous data showing that calpain-6 expressing CSCs are tumor-initiating cells [8]. The metastatic model consisting of IC injection of osteosarcoma cells indicates that calpain-6 expressing cells are also involved during the early stages of metastasis outgrowth.

We show that calpain-6 controls YAP stability by modulating the Hippo pathway and recycling of the β-catenin degradation complex. The underlying mechanisms were suggested by previous reports [23, 33–35] and confirmed here by the RNAseq analysis, which related calpain-6 to the organization of microtubule and actin cytoskeleton, autophagy, RNA splicing, endocytosis and with the “establishment of organelle localization”. These results validate the osteosarcoma cell models we used. They also highlight a new possible function of calpain-6 in the trafficking of intracellular vesicles. Together, they further posit this biomarker as a key controller of the cytoskeleton-dependent signaling in CSCs, especially Rho GTPase, Wnt and Hippo signaling.

Previously, in carcinoma mouse models, YAP was found involved in metastasis out-growth depending on cell-cell signaling [36]. Calpain-6 may also regulate the Hippo pathway and YAP transcriptional activity by modulating cell-cell interactions in sarcoma SCs because the suppression of calpain-6 with shRNA affected the expression of different genes coding cadherins such as CDH2 and CDH11. Finally, although YAP is a mechano-sensor whose activity is regulated in response to matrix characteristics, calpain-6 increased YAP activity independently of the physical properties of the microenvironment [37, 38]. This finding indicates that the CSCs have intrinsic advantages over other tumor cells to promote YAP activity in various environments.

Although calpain-6 and YAP levels were clearly correlated in cells and osteosarcoma tissues, we did not show significant enrichment of the large non-specific YAP signature in calpain-6 expressing sarcoma tissues. In contrast, we identified a G2M-restricted YAP activity that is promoted by calpain-6. Especially, we show that the calpain-6/YAP axis controls the expression of CENPF, TOP2A and ECT2. These proteins are important modulators of the cell cycle and cancer cell proliferation [39, 40]. They contribute to a prognosis signature in hepatocellular carcinoma and breast cancer [41, 42] and are associated with the development of metastases in synovial sarcoma and cervical cancer [43, 44]. TOP2A and ECT2 were found up-regulated in osteosarcomas associated with lung metastases as compared with non-metastatic bone tumors [45]. In contrast, we did not show any association between the expression of calpain-6 or mitotic genes and the outcome of patients with osteo- or soft-tissue sarcomas, which makes sense because calpain-6 expressing cells were the majority population in early steps of tumor outgrowth, whereas the CSC population was more or less sparse in mature mouse and human tumors and metastases. Hence, with our cell and mouse models, we revealed mechanisms that could be important during cancer initiation and relapses but may be difficult to identify in mature tissues that are deeply modified as compared with initial tumor buds. The disappearance/replacement of initial mechanisms in tumor over time could explain the failure to find efficient therapeutic targets to reduce the risk of metastasis in patients with sarcomas.

Calpain-6 expressing CSCs may contribute to the metastasis process also because of increased chemoresistance. We previously showed that chemoresistance in osteosarcomas is related to calpain-6 expression [46]. Our transcriptomic analyses further revealed that calpain-6 is associated with the expression of genes of G2M checkpoints and DNA repair. Because chemoresistance capacities via increased DNA repair were found a common CSC feature, calpain-6 may, thereby, be involved in the persistence of the disease after treatment [47, 48]. YAP was shown to contribute to the radiotherapy resistance of different types of tumors [49, 50]. This factor may therefore play a role in the CSC-associated chemoresistance. Further studies will determine whether inhibiting YAP activity could reverse CSC chemoresistance in sarcomas and the possible impact on disease recurrence.

Although verteporfin has anti-tumor effects by inducing of the death of actively dividing CSCs during early stages of tumor development, YAP inhibition resulted in increased metastasis development in mice with fast-growing tumors. This finding agrees with the consensus that YAP controls the size of progenitor cell populations but has opposing effects to regulate stem cell fate [12, 13, 51]. In contrast, our data show that the pro-tumoral effect of verteporfin can be via actions on the host tissues. YAP controls the resolution of inflammation in lungs [26]. Here we showed that verteporfin could further stimulate the tumor-related inflammation, leading to a vicious cycle in promoting proliferation of tumor cells. It will be of great interest to investigate why this inflammation was restricted to the tissue surrounding the lung nodules but did not affect tumor nodules because this may be related to tumor-dependent immunomodulation.

In conclusion, calpain-6 promotes a specific YAP-activity in CSCs and thus prevents death associated with intensive cell division to allow for tumor and metastasis out-growth. YAP inhibition could be an efficient strategy to prevent local or metastatic relapses, but the resulting effect will depend on the inflammatory context.

## Materials and methods

### Osteosarcoma cell lines and cell culture

Human 143B, U2OS and murine K7M2 osteosarcoma cell lines were obtained from ATCC (Manassas, VA, USA). The human and mouse cell lines were maintained in Dulbecco’s modified Eagle’s medium (Gibco) and RPMI medium 1640 (ThermoFisher scientific, Villebon, France), respectively. Culture medium was supplemented with 10% fetal calf serum (Sigma Aldrich, St Louis, MO, USA) and 100 IU/ml penicillin and 100 μg/ml streptomycin. The cells were treated to prevent mycoplasma contamination. The reporter Calp6-P-GFP plasmid was previously described [8]. The 143B cells expressing GFP under control of the calpain-6 regulatory sequence, were sorted by using the BD FACS ARIA II (BD Biosciences, San Jose, CA). Only cells with high GFP expression were selected. We transferred non-silencing or calpain-6-specific shRNA sequences that were previously described [46] into the lentiviral vector pLKO.1 puro a gift from Bob Weinberg (Addgene, Plasmid N°8453) [52]. Lentiviral particles were prepared in 293 T cells by using a packaging plasmid kit (Cellecta, Mountain View, CA, USA) and then mixed with medium containing 1 µl/ml polybrene for transduction. The cells that stably expressed control non-silencing shRNA were transduced to overexpress calpain-6 as described [9]. Alternatively, 143B cells were transduced with lentiviral particles to express a set of specific Calpain-6 shRNA (sc-62066-V, Santa Cruz Biotechnology, CA, USA). Murine Calp6-P-GFP K7M2 cells were transduced to express tomato under the EF1α promoter and luciferase with ready-to-use lentiviral particles (LV439, Amsbio, Abingdon, United Kingdom). For stable genomic integration of exogenous DNA, transduced cells were selected with G418, blasticidin or puromycin. To optimize calpain-6 and Calp6-P-GFP expression, cells were cultured in 3% O2 for 24 hr before analyses.

### RNA extraction and RT-PCR analyses

Total RNAs was extracted from osteosarcoma cells by using the ISOLATE II RNA Mini Kit (Bioline). Reverse transcription was performed with random primers with the High-Capacity cDNA Reverse Transcription Kit (Applied Biosystems). Real time quantitative PCR was then performed by using the primers listed in supplemental Table 1 and the SensiFAST™ SYBR® No-ROX Kit on the LightCycler 480 (Roche Applied Science, Indianapolis, IN, USA). ACTB and PPIA were housekeeping genes. Results are expressed as (2^∆*CT*^)*100, ∆*CT* = ((CT^*ACTB*^ - CT^*PPIA*^) /2) - CT^*x*^ or as Fold change in expression normalized by using the ΔΔCt formula.

### TEAD transcriptional activity

To test the effect of calpain-6 on YAP-dependent transcriptional activity, we used 8xGTIIC-luciferase construction, a gift from Stefano Piccolo (Addgene plasmid # 34615). Cells were cotransfected with 0.2 mg/well GT11c or control empty plasmid and 10 ng/well phRLSV40 (Renilla expression plasmid was an internal transfection control). At 48 hr after transfection, Firefly and Renilla luciferase activity was measured sequentially by using Luciferase Reporter Assay Systems (Promega). Luciferase activity was normalized both to Renilla activity, as a transfection control, and to values obtained with cells transfected with an empty plasmid. The cells were seeded on regular plastic plates or on collagen-coated silicon with controlled elastic modulus of 0.5, 2, 64 KPa (Cytosoft plates, Advanced BioMatrix, Carlsbad, CA, USA).

### RNA sequencing (RNA-Seq)

RNA purity/quality (RIN ≥ 7) was assessed with the 2100 Agilent Bioanalyzer by using the Agilent small RNA kit. RNAseq involved using the Integragen platform. Libraries were prepared with the Ultra II Directional RNA Library Prep Kit for Illumina protocol according to the supplier recommendations. Briefly, the key stages of this protocol are successive for the purification of PolyA containing mRNA molecules using poly-T oligo attached magnetic beads from 1 µg total RNA (with the Magnetic mRNA Isolation Kit from NEB), fragmentation by using divalent cations under elevated temperature to obtain approximately 300 bp pieces, double-strand cDNA synthesis and finally Illumina adapters ligation and cDNA library amplification by PCR for sequencing. Sequencing was then carried out on Paired End 100b reads of Illumina NovaSeq. Image analysis and base calling involved using Illumina Real Time Analysis v3.4.4 with default parameters.

### RNA-Seq data processing

A computational pipeline for RNA-Seq data was established for analyzing data from the identified experiments in a high-throughput manner. All datasets were analyzed with the same pipeline. Quality of raw sequencing reads was assessed using FastQC v0.11.9 to estimate the proportions of reads of low quality. Reads were aligned on the reference genome by using Kallisto v0.46.1. The Kallisto index was used for building up the reference genomes for reads mapping. The transcriptomic coordination of *Homo sapiens* (GRCh38.p13) downloaded from Ensembl (release 99) was translated to filter out alignments carrying insertions or deletions or falling outside the identified transcriptome regions. To quantify the expression, Kallisto was used for abundance estimates of paired end samples in transcripts per million (TPM) [53]. Read count matrices for each cell sample were produced with Tximport v1.14.2 [54]. The abundance quantification with Tximport identifies the expected counts for each gene. The per-gene counts were given as input for gene differential expression analysis using DESeq2 v1.26.0 with default settings [55]. Genes with log2 fold changes > 1 and < −1 were identified as significantly up- and down-regulated, respectively. To obtain insight into the potential functions of differentially expressed genes, Gene Ontology (GO) and Kyoto Encyclopedia of Genes and Genomes (KEGG) enrichments were obtained by using ClusterProfiler v3.14.3. GO analysis involved using the enrichGO function in ClusterProfiler with settings ont = “BP”, pAdjustMethods = “BH”, pvalueCutoff= 0.001, qvalueCutoff = 1. KEGG analysis was conducted using the enrichKEGG function in ClusterProfiler for pathway representations with settings organism = “hsa”, pvalueCutoff = 0.05, qvalueCutoff = 1. To investigate the molecular details of specific biological pathways, the Reactome Pathway Analysis (ReactomePA) function implemented in ClusterProfiler was used to explore the connections between experimental information and well-annotated established pathway data, focusing on intermediary metabolism. An adjusted *p* < 0.05 was considered as significant and was calculated based on the Benjamini-Hochberg procedure [56].

### Gene set enrichment analysis (GSEA)

Two datasets were downloaded from TCGA (https://portal.gdc.cancer.gov) to analyze the relation between CAPN6 and YAP expression in bone (Target-OS) and soft-tissue sarcomas (TCGA-Sarc) [57]. The RNA-Seq profiles of the tumors and the 143B cells were analyzed with the GSEA software [58]. The gene sets are listed in supplemental Table 2. The enrichment scores were based on a FDR < 0.25 after 1000 permutations.

### Immunoblots

Cells were washed with cold phosphate buffered saline (PBS) and then lysed on ice in 50 mM TRIS buffer (pH 7.5) containing 150 mM NaCl, 1% NP-40, 10% glycerol and protease inhibitors. Proteins lysates were subjected to SDS-PAGE, electro-transferred on a PVDF membrane, then incubated with the primary antibodies and appropriate HRP-conjugated secondary antibodies. The signals were visualized by using a chemiluminescent detection system (Azure Spectra). Bands were quantified by using ImageJ. The primary antibodies are listed in Supplemental Table 3.

### Immunohistochemistry

Mouse bone tumors and lungs were fixed in 4% PFA in PBS, then paraffin-embedded. After dewaxing, 5-µm tissue sections were incubated at 70 °C in citrate buffer, pH6, for 4 hr. The tissues were then blocked for endogenous peroxidase activity with 3% H_2_0_2_ in PBS and for non-specific Ig coupling with PBS containing 0.02% Tween-20, 2.5% serum horse, 2.5% bovine serum albumin (BSA). The tissue sections were incubated overnight at 4 °C with primary antibodies, then with HRP-conjugated secondary Ig. The ImPRESS HRP polymer detection kit (Vector) with DAB substrate was used to reveal specific staining. The tissue sections were counterstained with purified methyl green. The sections were then dehydrated with xylene and mounted by using Entellan mounting (Merck, Guyancourt, France). Goat, rabbit or mouse IgG replaced specific primary antibodies to serve as negative controls. The primary antibodies are listed in supplemental Table 3.

### Immunofluorescence staining

Immunocytochemistry was performed after cell fixation with 4% PFA in PBS and saturation of the non-specific sites with BSA and donkey serum. The cells were permeabilized by adding Igepal or Triton X-100 in the saturation solution. For immunohistochemistry we collected paraffin-embedded specimens of resected pulmonary metastatic tissue from 3 patients with well-characterized osteosarcoma. Informed consent was obtained from each patient or the patient’s guardian. Sections from these tissues or 143B cells were used to analyze YAP and calpain-6 expression. Saturation of non-specific Ig binding sites involved using PBS containing 0.02% Triton-X100, 1% donkey serum, 1% BSA. Secondary antibodies consisted of DyLight-550 or -480-conjugated anti- IgG (Thermo Fisher Scientific). Nuclei were then counterstained with DAPI at 0.1 µg/ml. To reduce autofluorescence in tissue sections, we used the TRUEVIEW quenching kit (Vector, Burlingame, CA, USA). The primary antibodies are listed in supplemental Table 3.

### Image acquisitions and processing

Image were acquired by using the ApoTome optical sectioning system (Zeiss, Paris, France) with an inverted microscope (Zeiss Axio Observer Z1) for immunofluorescent images and a Nikon microscope (type 120c) for chromogenic images. Zenpro (Zeiss) and Adobe Photoshop were used for image processing. For comparing of fluorescence images, contrast and brightness were adjusted identically. All other quantifications involved using ImageJ.

### Flow cytometry analyses

For intracellular staining, cells were fixed and permeabilized by using the Fix-Perm kit (Thermo Fisher Scientific) before incubation with antibodies (supplemental Table 3). To assess cell division, the PKH26 Red Fluorescent Cell Linker Kit was used as a membrane marker at the onset of the culture (Sigma Aldrich). Fluorescence analysis was performed in FACS CANTO II with Diva software (Becton Dickinson). A classical gating strategy using FSC-A versus SSC-A and FSC-A versus FSC-H was used to select cells for the analysis (Supplemental information).

### Mouse osteosarcoma models

S/SPF BALB/cByJ mice were purchased from Charles River Breeding Laboratories. They were 6 weeks old males. Mice were kept in accordance with the institutional guidelines of the French Ethical Committee and under the supervision of authorized investigators. In vivo experiments were approved by the Local Ethical Committee (APAFIS #26491). K7M2 cells were cultured for 24 hr in 3% O2, collected and counted. Fifty thousand cells in 5 µl PBS were injected into the right tibia of the mice as previously described [59]. Briefly, the mice were anesthetized with a Ketamine/Xylasine mix (100/50 mg/kg) and received preventive analgesia with subcutaneous injections of 50 µg/kg of buprenorphine. The right tibia was drilled and a needle was inserted to slowly inject the cell suspension. Verteporfin (Sigma Aldrich) was administrated at 6 mg/kg via intraperitoneal injections. The injection plan is in Supplementary Fig. 7. Mice were treated twice a week for 8 weeks, with no significant modification of weight. DMSO diluted in PBS was administered to control mice. Bone tumor growth was monitored longitudinally in live animals every week either by measuring the tumor with a Vernier caliper or using bioluminescence imaging. Animals were sacrificed when the tumor reached 1 cm in diameter. The Perkin Elmer’s ProSense-680 probe was used to assess inflammation in tumor-developing mice. This is a protease activatable NIR fluorescent probe that is optically silent in its intact state and becomes highly fluorescent after cathepsin-mediated cleavage.

### Tumor growth monitoring by optical imaging

Mice were anesthetized by isofluorane inhalation (0.9 mg per mouse) and positioned under a cooled intensified charge-coupled device (CCDi) camera (Biospace,) with the injected tibia stretched out. Before the acquisition, 2 mg of luciferin potassium salt (D-luciferin, Interchim) diluted in PBS was injected intraperitoneally. Bioluminescent signals were acquired for 10 min by using the Photo Acquisition software (BiospaceLab). The bioluminescence intensity (BLI) was measured in the region of interest (2 cm^2^ ROI) corresponding to the tumor area by using the M3Vision+ software (Biospace). BLI is the total radiant efficiency (photons/second/sr) of the tumor corrected with the radiant efficiency of an international positive control LED.

### Statistics

Statistical analysis was performed with GraphPad Prism v9.2.0. Differences between 2 groups were tested with two-tailed Student *t* tests and for multiple comparisons with one-way oor two-way ANOVA. The statistical tests were performed on biological replicates. Excepted each experiment was repeated independently 2–3 times and results were pooled when possible. The RNAseq analysis of Calp6-P-GFP cells, Calp6+ cells and Calp6 shRNA cells was performed by using RNA extracted from 3 independent cultures for each condition. Correlations were tested using simple linear regressions.

## Supplementary information

Supplementary informations

Supllementary figures

Original Data File

checklist

## Data Availability

The datasets generated during the current study are available at the following links: 10.5061/dryad.z34tmpghd. The other datasets used and/or analyzed during the current study are available from the corresponding author on reasonable request.

## References

[1] Doyle LA (2014). Sarcoma classification: An update based on the 2013 World Health Organization Classification of Tumors of Soft Tissue and Bone. Cancer.

[2] Allemani C, Weir HK, Carreira H, Harewood R, Spika D, Wang X-S (2015). Global surveillance of cancer survival 1995-2009: analysis of individual data for 25,676,887 patients from 279 population-based registries in 67 countries (CONCORD-2). Lancet (Lond, Engl).

[3] Krishnamoorthy N, Desai SS, Rekhi B, Jambhekar NA (2011). A clinico-morphological study of 95 cases of sarcomas with metastases to the lungs. Indian J Cancer.

[4] Gambera S, Abarrategi A, González-Camacho F, Morales-Molina Á, Roma J, Alfranca A, et al. Clonal dynamics in osteosarcoma defined by RGB marking. Nat Commun. 2018. https://pubmed-ncbi-nlm-nih-gov.proxy.insermbiblio.inist.fr/30266933/10.1038/s41467-018-06401-z PMC6162235 30266933

[5] Hotfilder M, Mallela N, Seggewiß J, Dirksen U, Korsching E (2018). Defining a Characteristic Gene Expression Set Responsible for Cancer Stem Cell-Like Features in a Sub-Population of Ewing Sarcoma Cells CADO-ES1. Int J Mol Sci.

[6] Gibbs CP, Kukekov VG, Reith JD, Tchigrinova O, Suslov ON, Scott EW (2005). Stem-like cells in bone sarcomas: implications for tumorigenesis. Neoplasia [Internet].

[7] Nandy SB, Lakshmanaswamy R Cancer Stem Cells and Metastasis. In: Progress in Molecular Biology and Translational Science. Prog Mol Biol Transl Sci. 2017. https://pubmed-ncbi-nlm-nih-gov.proxy.insermbiblio.inist.fr/29096892/10.1016/bs.pmbts.2017.07.007 29096892

[8] Andrique C, Morardet L, Linares LK, Cissé MY, Merle C, Chibon F, et al. Calpain-6 controls the fate of sarcoma stem cells by promoting autophagy and preventing senescence. JCI insight. 2018. https://insight.jci.org/articles/view/121225 10.1172/jci.insight.121225 PMC6171816 30185659

[9] Marion A, Dieudonné F-X, Patiño-Garcia A, Lecanda F, Marie PJ, Modrowski D (2012). Calpain-6 is an endothelin-1 signaling dependent protective factor in chemoresistant osteosarcoma. Int J Cancer.

[10] Liu Y, Mei C, Sun L, Li X, Liu M, Wang L (2011). The PI3K-Akt pathway regulates calpain 6 expression, proliferation, and apoptosis. Cell Signal.

[11] Varelas X (2014). The hippo pathway effectors TAZ and YAP in development, homeostasis and disease. Dev.

[12] Heng BC, Zhang X, Aubel D, Bai Y, Li X, Wei Y, et al. Role of YAP/TAZ in Cell Lineage Fate Determination and Related Signaling Pathways [Internet]. Vol. 8, Frontiers in Cell and Developmental Biology. Front Cell Dev Biol. 2020. https://pubmed-ncbi-nlm-nih-gov.proxy.insermbiblio.inist.fr/32850847/10.3389/fcell.2020.00735 PMC7406690 32850847

[13] Cao X, Wang C, Liu J, Zhao B Regulation and functions of the Hippo pathway in stemness and differentiation [Internet]. Vol. 52, Acta Biochimica et Biophysica Sinica. Acta Biochim Biophys Sin (Shanghai). 2021. https://pubmed-ncbi-nlm-nih-gov.proxy.insermbiblio.inist.fr/32445460/10.1093/abbs/gmaa048 32445460

[14] Li HL, Li QY, Jin MJ, Lu CF, Mu ZY, Xu WY, et al. A review: hippo signaling pathway promotes tumor invasion and metastasis by regulating target gene expression. J Cancer Res Clin Oncol. 2021. https://pubmed-ncbi-nlm-nih-gov.proxy.insermbiblio.inist.fr/33864521/10.1007/s00432-021-03604-8 PMC11801896 33864521

[15] Deel MD, Li JJ, Crose LES, Linardic CM A review: Molecular aberrations within Hippo signaling in bone and soft-tissue sarcomas. Front Oncol. 2015. https://pubmed-ncbi-nlm-nih-gov.proxy.insermbiblio.inist.fr/26389076/10.3389/fonc.2015.00190 PMC4557106 26389076

[16] Zhang YH, Li B, Shen L, Shen Y, Chen XD (2013). The role and clinical significance of yes-associated protein 1 in human osteosarcoma. Int J Immunopathol Pharm.

[17] Bouvier C, Macagno N, Nguyen Q, Loundou A, Jiguet-Jiglaire C, Gentet J-C (2016). Prognostic value of the Hippo pathway transcriptional coactivators YAP/TAZ and β1-integrin in conventional osteosarcoma. Oncotarget.

[18] Rhodes DR, Kalyana-Sundaram S, Mahavisno V, Varambally R, Yu J, Briggs BB (2007). Oncomine 3.0: Genes, pathways, and networks in a collection of 18,000 cancer gene expression profiles. Neoplasia.

[19] Basu-Roy U, Sumru Bayin N, Rattanakorn K, Han E, Placantonakis DG, Mansukhani A, et al. ARTICLE Sox2 antagonizes the Hippo pathway to maintain stemness in cancer cells. Nat Commun. 2015. www.nature.com/naturecommunications 10.1038/ncomms7411 PMC4429898 25832504

[20] Nie P, Li Y, Suo H, Jiang N, Yu D, Fang B (2019). Dasatinib Promotes Chondrogenic Differentiation of Human Mesenchymal Stem Cells via the Src/Hippo-YAP Signaling Pathway. ACS Biomater Sci Eng.

[21] Taelman VF, Dobrowolski R, Plouhinec JL, Fuentealba LC, Vorwald PP, Gumper I (2010). Wnt signaling requires sequestration of Glycogen Synthase Kinase 3 inside multivesicular endosomes. Cell.

[22] Gargini R, Escoll M, García E, García-Escudero R, Wandosell F, Antón IM (2016). WIP Drives Tumor Progression through YAP/TAZ-Dependent Autonomous Cell Growth. Cell Rep.

[23] Tonami K, Kurihara Y, Aburatani H, Uchijima Y, Asano T, Kurihara H (2007). Calpain 6 is involved in microtubule stabilization and cytoskeletal organization. Mol Cell Biol.

[24] Pattschull G, Walz S, Gründl M, Schwab M, Rühl E, Baluapuri A (2019). The Myb-MuvB Complex Is Required for YAP-Dependent Transcription of Mitotic Genes. Cell Rep.

[25] Deng Y, Lu J, Li W, Wu A, Zhang X, Tong W, et al. Reciprocal inhibition of YAP/TAZ and NF-κB regulates osteoarthritic cartilage degradation. Nat Commun. 2018 https://pubmed-ncbi-nlm-nih-gov.proxy.insermbiblio.inist.fr/30385786/10.1038/s41467-018-07022-2 PMC6212432 30385786

[26] Liu LY, Shan XQ, Zhang FK, Fan XF, Fan JM, Wang YY (2020). YAP activity protects against endotoxemic acute lung injury by activating multiple mechanisms. Int J Mol Med.

[27] LaCanna R, Liccardo D, Zhang P, Tragesser L, Wang Y, Cao T (2019). Yap/Taz regulate alveolar regeneration and resolution of lung inflammation. J Clin Invest.

[28] Couturier CP, Ayyadhury S, Le PU, Nadaf J, Monlong J, Riva G, et al. Single-cell RNA-seq reveals that glioblastoma recapitulates a normal neurodevelopmental hierarchy. Nat Commun. 2020. https://pubmed-ncbi-nlm-nih-gov.proxy.insermbiblio.inist.fr/32641768/10.1038/s41467-020-17186-5 PMC7343844 32641768

[29] Pan XW, Zhang H, Xu D, Chen JX, Chen WJ, Gan SS (2020). Identification of a novel cancer stem cell subpopulation that promotes progression of human fatal renal cell carcinoma by single-cell RNA-seq analysis. Int J Biol Sci.

[30] Liu Y, Feng W, Dai Y, Bao M, Yuan Z, He M, et al. Single-Cell Transcriptomics Reveals the Complexity of the Tumor Microenvironment of Treatment-Naive Osteosarcoma. Front Oncol. https://pubmed-ncbi-nlm-nih-gov.proxy.insermbiblio.inist.fr/34367994/10.3389/fonc.2021.709210 PMC8335545 34367994

[31] Zhou Y, Yang D, Yang Q, Lv X, Huang W, Zhou Z, et al. Single-cell RNA landscape of intratumoral heterogeneity and immunosuppressive microenvironment in advanced osteosarcoma. Nat Commun. 2020. https://pubmed-ncbi-nlm-nih-gov.proxy.insermbiblio.inist.fr/33303760/10.1038/s41467-020-20059-6 PMC7730477 33303760

[32] Nagasawa S, Kashima Y, Suzuki A, Suzuki Y (2021). Single-cell and spatial analyses of cancer cells: toward elucidating the molecular mechanisms of clonal evolution and drug resistance acquisition. Inflamm Regen [Internet].

[33] Tonami K, Kurihara Y, Arima S, Nishiyama K, Uchijima Y, Asano T (2011). Calpain-6, a microtubule-stabilizing protein, regulates Rac1 activity and cell motility through interaction with GEF-H1. J Cell Sci.

[34] Hong JM, Teitelbaum SL, Kim T-H, Ross FP, Kim S-Y, Kim H-J (2011). Calpain-6, a target molecule of glucocorticoids, regulates osteoclastic bone resorption via cytoskeletal organization and microtubule acetylation. J Bone Min Res.

[35] Miyazaki T, Tonami K, Hata S, Aiuchi T, Ohnishi K, Lei X-F (2016). Calpain-6 confers atherogenicity to macrophages by dysregulating pre-mRNA splicing. J Clin Invest [Internet].

[36] Er EE, Valiente M, Ganesh K, Zou Y, Agrawal S, Hu J (2018). Pericyte-like spreading by disseminated cancer cells activates YAP and MRTF for metastatic colonization. Nat Cell Biol.

[37] Cai X, Wang KC, Meng Z. Mechanoregulation of YAP and TAZ in Cellular Homeostasis and Disease Progression. Front Cell Dev Biol. 2021. 10.3389/fcell.2021.673599 PMC8182050 34109179

[38] Pavel M, Renna M, Park SJ, Menzies FM, Ricketts T, Füllgrabe J, et al. Contact inhibition controls cell survival and proliferation via YAP/TAZ-autophagy axis. Nat Commun. 2018. https://pubmed-ncbi-nlm-nih-gov.proxy.insermbiblio.inist.fr/30054475/10.1038/s41467-018-05388-x PMC6063886 30054475

[39] Varis A, Salmela AL, Kallio MJ (2006). Cenp-F (mitosin) is more than a mitotic marker [Internet]. Chromosom Chromosom.

[40] Tao Q, Chen S, Liu J, Zhao P, Jiang L, Tu X (2021). The roles of the cell division cycle-associated gene family in hepatocellular carcinoma. J Gastrointest Oncol.

[41] Huang Y, Chen X, Wang L, Wang T, Tang X, Su X (2021). Centromere protein F (CENPF) serves as a potential prognostic biomarker and target for human hepatocellular carcinoma. J Cancer.

[42] Sun J, Huang J, Lan J, Zhou K, Gao Y, Song Z, et al. Overexpression of CENPF correlates with poor prognosis and tumor bone metastasis in breast cancer. Cancer Cell Int. 2019;19. 10.1186/s12935-019-0986-8 PMC6788011 31632198

[43] Y S, X L, F W, X W, G C, C P. Identification of Metastasis-Associated Biomarkers in Synovial Sarcoma Using Bioinformatics Analysis. Front Genet. 2020. https://pubmed-ncbi-nlm-nih-gov.proxy.insermbiblio.inist.fr/33061942/10.3389/fgene.2020.530892 PMC7518102 33061942

[44] Yu B, Chen L, Zhang W, Li Y, Zhang Y, Gao Y, et al. TOP2A and CENPF are synergistic master regulators activated in cervical cancer. BMC Med Genomics. 2020. https://pubmed-ncbi-nlm-nih-gov.proxy.insermbiblio.inist.fr/33023625/10.1186/s12920-020-00800-2 PMC7541258 33023625

[45] Liu D, Zhou R, Zhou A (2021). Identification of key biomarkers and functional pathways in osteosarcomas with lung metastasis: Evidence from bioinformatics analysis. Med (Baltim) [Internet].

[46] Marion A, Dieudonné F-X, Patiño-Garcia A, Lecanda F, Marie PJ, Modrowski D (2012). Calpain-6 is an endothelin-1 signaling dependent protective factor in chemoresistant osteosarcoma. Int J Cancer.

[47] Hein AOuellete MYan Y (2014). Radiation-induced signaling pathways that promote cancer cell survival (review). Int J Oncol.

[48] Abad E, Graifer D, Lyakhovich A. DNA damage response and resistance of cancer stem cells. Vol. 474, Cancer Letters. Cancer Lett. 2020https://pubmed-ncbi-nlm-nih-gov.proxy.insermbiblio.inist.fr/31968219/10.1016/j.canlet.2020.01.008 31968219

[49] Akervall J, Nandalur S, Zhang J, Qian CN, Goldstein N, Gyllerup P (2014). A novel panel of biomarkers predicts radioresistance in patients with squamous cell carcinoma of the head and neck. Eur J Cancer.

[50] Ghiso E, Migliore C, Ciciriello V, Morando E, Petrelli A, Corso S (2017). YAP-Dependent AXL Overexpression Mediates Resistance to EGFR Inhibitors in NSCLC. Neoplasia (U S).

[51] Zhang X, Abdelrahman A, Vollmar B, Zechner D The ambivalent function of YAP in apoptosis and cancer [Internet]. Vol. 19, International Journal of Molecular Sciences. Int J Mol Sci; 2018. https://pubmed-ncbi-nlm-nih-gov.proxy.insermbiblio.inist.fr/30486435/10.3390/ijms19123770 PMC6321280 30486435

[52] Stewart SA, Dykxhoorn DM, Palliser D, Mizuno H, Yu EY, An DS (2003). Lentivirus-delivered stable gene silencing by RNAi in primary cells. RNA.

[53] Bray NL, Pimentel H, Melsted P, Pachter L (2016). Near-optimal probabilistic RNA-seq quantification. Nat Biotechnol.

[54] Soneson C, Love MI, Robinson MD Differential analyses for RNA-seq: Transcript-level estimates improve gene-level inferences. F1000Research. 2016;4. Available from: https://pubmed-ncbi-nlm-nih-gov.proxy.insermbiblio.inist.fr/26925227/10.12688/f1000research.7563.1 PMC4712774 26925227

[55] RC G, VJ C, DM B, B B, M D, S D, et al. Bioconductor: open software development for computational biology and bioinformatics. Genome Biol. 2004;5. 10.1186/gb-2004-5-10-r80 PMC545600 15461798

[56] Boyle EI, Weng S, Gollub J, Jin H, Botstein D, Cherry JM (2004). GO::TermFinder - Open source software for accessing Gene Ontology information and finding significantly enriched Gene Ontology terms associated with a list of genes. Bioinforma.

[57] Grossman RL, Heath AP, Ferretti V, Varmus HE, Lowy DR, Kibbe WA (2016). Toward a Shared Vision for Cancer Genomic Data. N. Engl J Med.

[58] Subramanian A, Tamayo P, Mootha VK, Mukherjee S, Ebert BL, Gillette MA (2005). Gene set enrichment analysis: A knowledge-based approach for interpreting genome-wide expression profiles. Proc Natl Acad Sci USA.

[59] Dieudonné F-X, Marion A, Marie PJ, Modrowski D (2012). Targeted inhibition of T-cell factor activity promotes syndecan-2 expression and sensitization to doxorubicin in osteosarcoma cells and bone tumors in mice. J Bone Miner Res.

