# Supplementary material for: A calpain-6/YAP axis in sarcoma stem cells that drives the outgrowth of tumors and metastases
Source: Cell Death Dis. 2022 Sep 24;13(9):819. doi: 10.1038/s41419-022-05244-3 (PMC9509353; doi:10.1038/s41419-022-05244-3)
Supplement: Supplementary file 1 — Supplementary informations [file 41419_2022_5244_MOESM1_ESM.pdf]

Supplementary information

Table 1: Primers

	Forward	Reverse	Supplier
ANKRD1	5'-AGTAGAGGAACTGGTCACTGGT-3'	5'-TGGGCTAGAAGTGTCTTCAGA-3'	Eurogentec
AXL	5'-CACCAGCAAGAGCGATGTGT	5'-CGGTCCTGGGGATTTAGCTC-3'	Eurogentec
CTGF	5'-AGGAGTGGGTGTGTGACGA-3'	5'-CCAGGCAGTTGGCTCTAATC-3'	Eurogentec
Cyr61	5'-GCCAACCAGCATTCCTGAGA-3'	5'-GAGCCCGCCTTTTATACGGG-3'	Eurogentec
YAP	5'-GCACCTCTGTGTTTTAAGGGTCT-3'	5'-CAACTTTTGCCCTCCTCCAA-3'	Eurogentec
actine	5'-GAGAAGAGCTACGAGCTGCCTG-3'	5'-GGTAGTTTCGTGGATGCCACA-3'	Eurogentec
PPia	5'-GTCAACCCCACCGTGTTCTT-3'	5'-CTGCTGTCTTTGGGACCTTGT-3'	Eurogentec

Table 2: Gene sets

CORDENONSI_YAP_CONSERVED_SIGNATURE

GO:1902751, positive regulation of cell cycle G2/M phase transition

REACTOME_G2_M_CHECKPOINTS

HALLMARK_DNA_REPAIR

YAP-dependent mitotic gene signature from Pattschull et al., 2019: (Entrez ID) 259266, 991, 1063, 283431, 3161, 51203, 7153, 890, 6790, 1031, 2621, 7052, 5054, 7057, 332, 29969, 4082, 3925, 26227, 3268, 1894

Table 3: Antibodies

Antibodies				
Protein	host	Supplier	reference	Dilution
Calpain-6	Rabbit	Cusabio	CSB-PA897594LA01HV	1:10000 (WB)
				1:2500 (IF)
Actin	Rabbit	Sigma-Aldrich		1:1000 (WB)
YAP	Mouse	Santa-Cruz	sc-101199	1:1000 (WB)
				1 :200 (IF)
TAZ	Mouse	BDBiosciences	560235	1:1000 (WB)
P-lats (serine 127)	Rabbit	Cell Signaling	9157S	1:1000 (WB)
Lats1 (N-term)	Rabbit	Sigma	SAB1300096	1:1000 (WB)
β-catenin	Mouse	Santa-Cruz	sc-7963	1:1000 (WB)
GSK3 α/β	Mouse	Santa-Cruz	sc-7291	1:1000 (WB) 1:250 (IF)
AXIN	Rabbit	Invitrogen	34-5900	1:1000 (WB)
α -tubulin	Mouse	Santa-Cruz	sc-51500	1:1000 (IF)
GFP-FITC	GOAT	ABCAM	Ab6662	1 :1000 (IF)
P-YAP (serine127)	Rabbit	Cell Signaling	4911S	1:1000 (WB)
Ki-67 (clone SP6)	Rabbit	Zytomed Systems	Z2031RS	1 :100 (IHC)
NFKB p65 (G-8)	Mouse	Santa-Cruz	Sc-398442	1 : 100 (IHC)

Supplementary information 4: Flow cytometry: Example for the gating strategy Calp6-P-GFP 143B cells were fixed, permeabilized and stained for YAP1

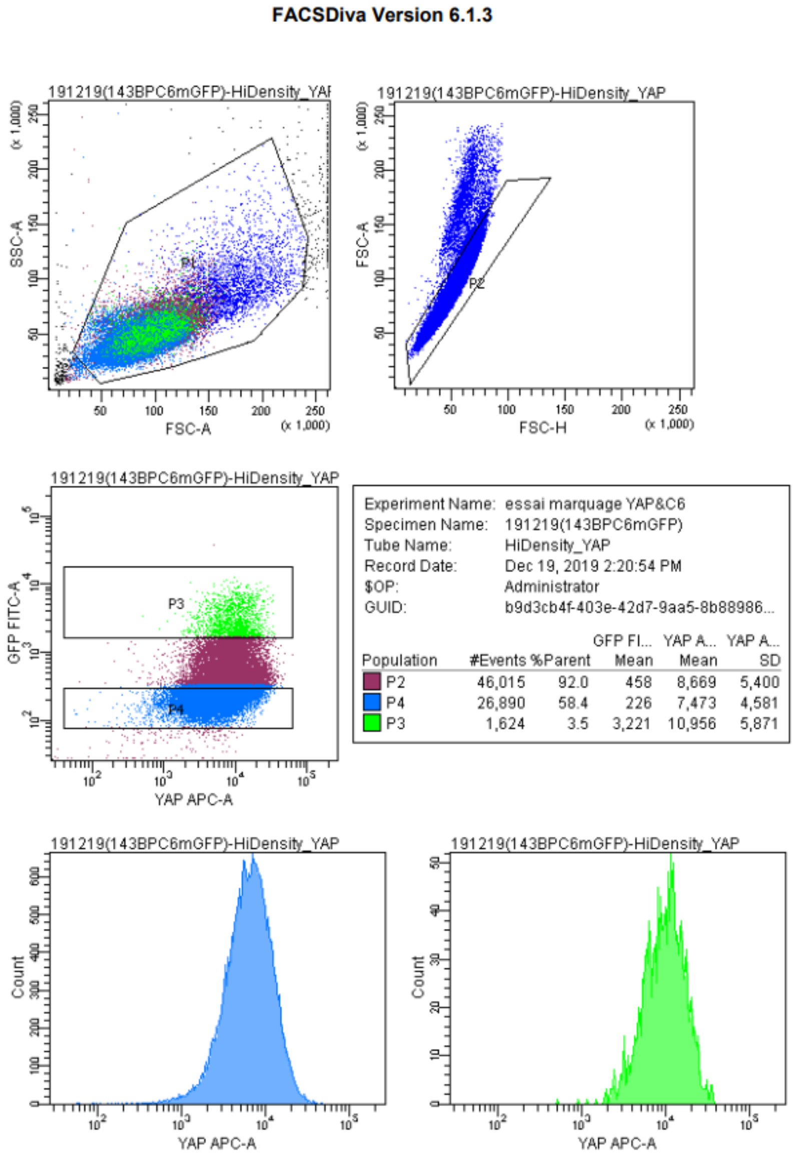

P1 gate selected alive cells

P2 selected single cells

P3 selected GFP+ cells & P4 GFP negative cells

Panels at the bottom of the figure show Yap staining in GFP- (left) and GFP+ (right) cells.
