# Supplementary material for: A calpain-6/YAP axis in sarcoma stem cells that drives the outgrowth of tumors and metastases
Source: Cell Death Dis. 2022 Sep 24;13(9):819. doi: 10.1038/s41419-022-05244-3 (PMC9509353; doi:10.1038/s41419-022-05244-3)
Supplement: Supplementary file 2 — Supllementary figures [file 41419_2022_5244_MOESM2_ESM.pdf]

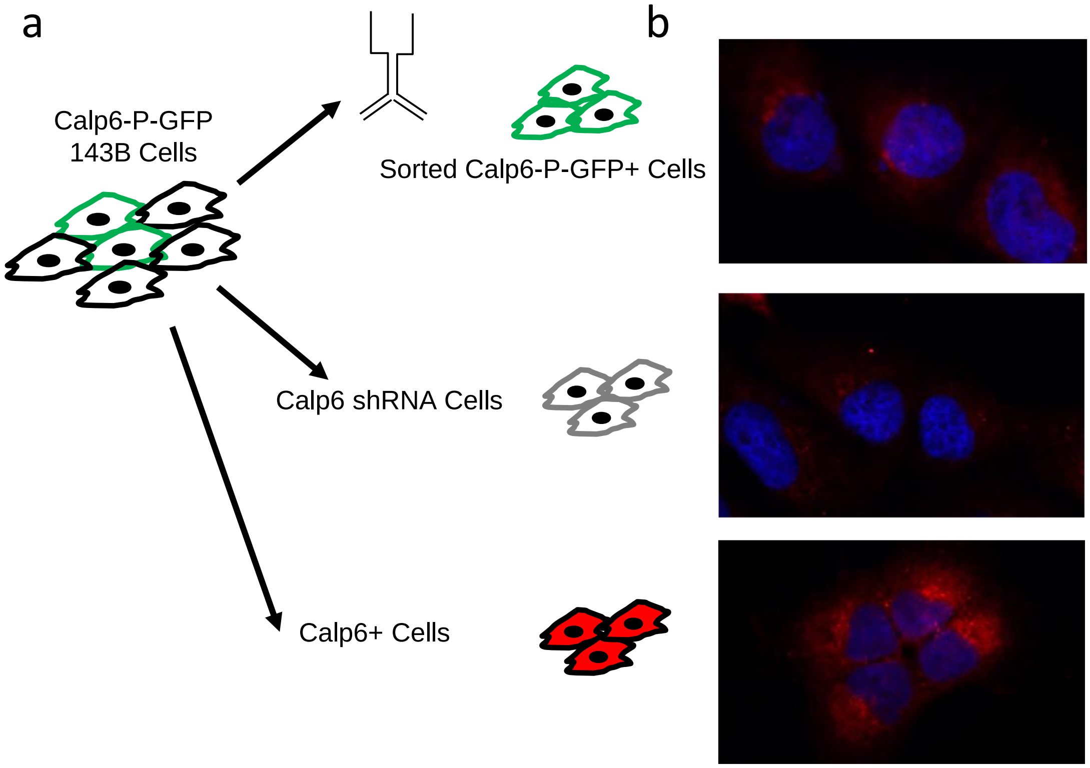

Supplementary Figure 1: a, 143B cells were modified to express GFP under the control of CAPN6 regulatory sequence (Calp6-P-GFP Cells). The Calp6-P-GFP+ cells were sorted to isolate cells that express basal levels of calpain-6. Calp6-P-GFP cells were transduced with PLKO-puro-shRNA to suppress calpain-6 expression (Calp6 shRNA Cells). Calp6-P-GFP cells expressing control non-silencing shRNA were transduced with a lentiviral vector to overexpress calpain-6 (Calp6+ Cells). RNA were extracted from these different cell populations to performed the transcriptomic analysis. b, Immunofluorescence of calpain-6 (red) in Calp6-P-GFP+ cells (upper panel), Calp6 shRNA expressing cells (middle panel), Calpain-6 overexpressing cells (bottom panel). DAPI served to stain nuclei.

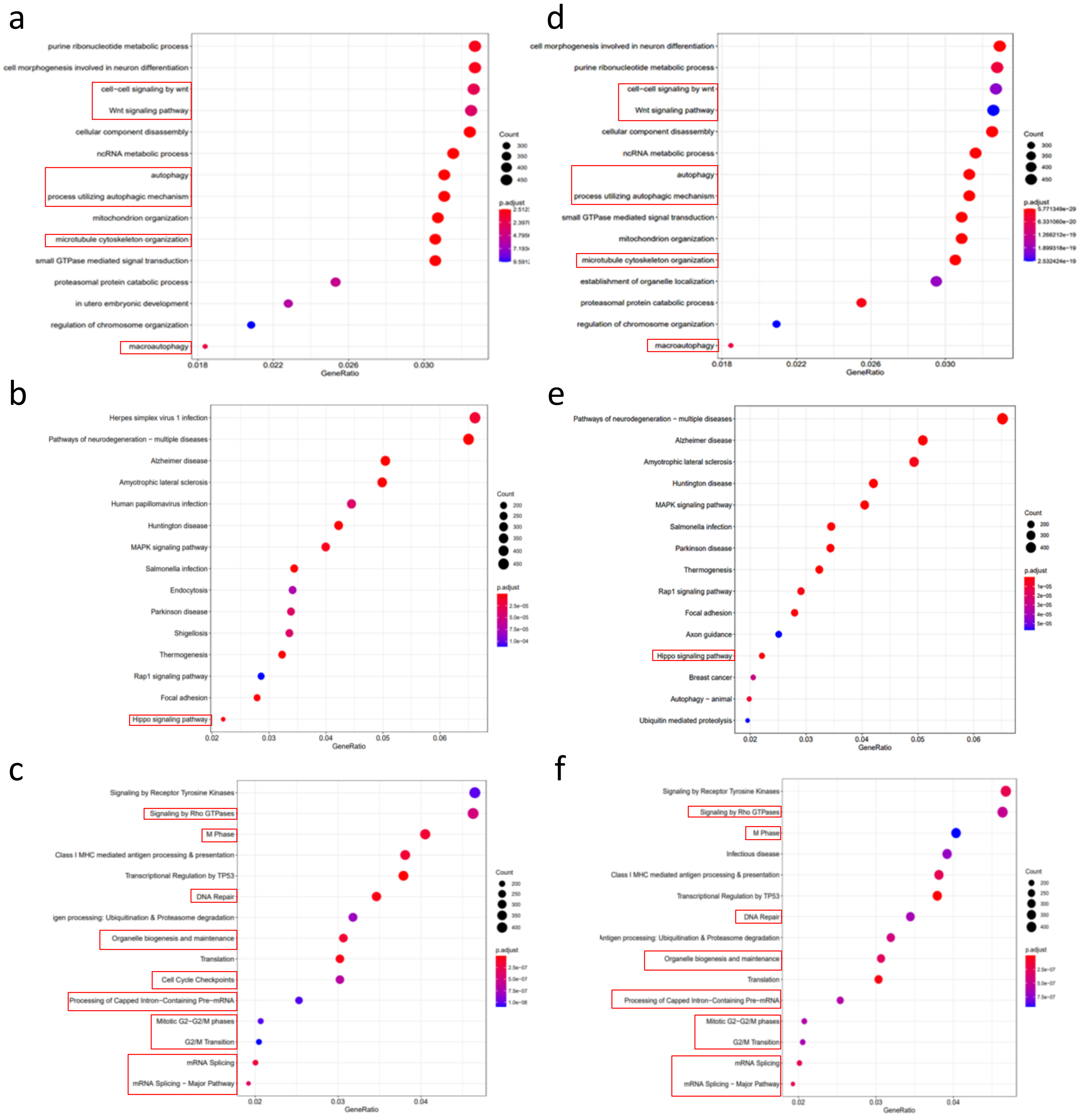

Supplementary Figure 2: **Analyses of the potential functions of differentially expressed genes** in Calp6-P-GFP+ vs Calp6 shRNA cells (a, b, c), and Calp6+ vs Calp6 shRNA cells (d, e, f) using Gene Ontology (a, d), Kyoto Encyclopedia of Genes (b,e) and Reactome Pathway Analysis (c, f). The size of the dots indicates the number of genes of the signature differentially expressed (count) and the p values are indicated with the color code (p. adjust). Red frames show pathways that are discussed in the text of the article.

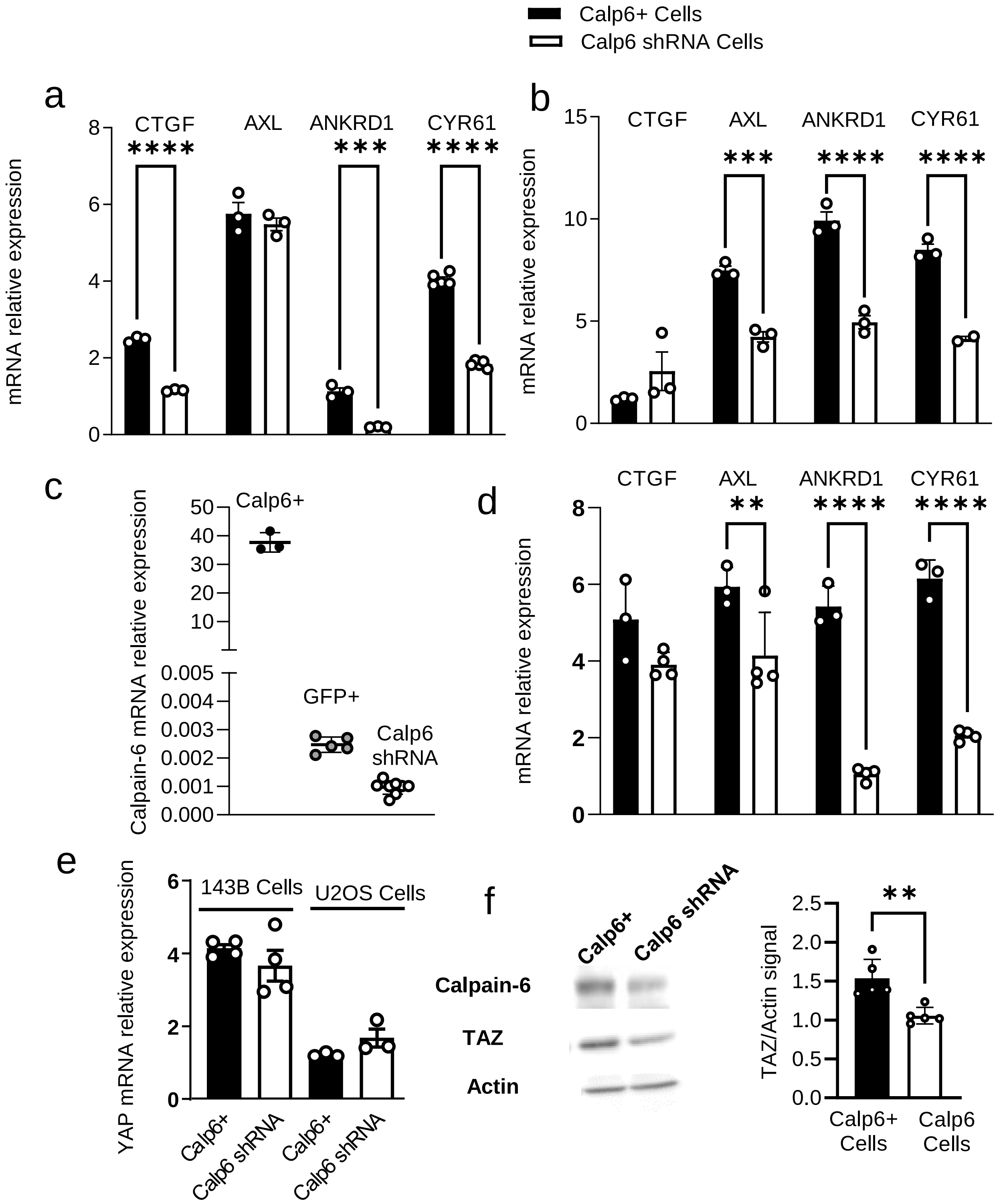

Supplementary Figure 3 : **Increased YAP activity was associated with YAP and TAZ protein accumulation.** Expression of YAP/TEAD targets as indicated in calpain-6 overexpressing (Calp6+) and calpain-6 shRNA expressing (Calp6 shRNA) 143B (a) or U2OS (b) cells. c,d, Effect of a different set of control and calpain-6 shRNA on the expression of calpain-6 (c) and YAP/TEAD targets genes (d) as compared to calpain-6 overexpressing cells (Calp6+). e, Expression of YAP mRNA in Calp6+ vs Calp6 shRNA 143B and U2OS cells. Results are expressed as mean % of ACT/PPIA expression ± SEM. f, Western blot analysis of TAZ protein in Calp6+ and Calp6 shRNA 143B cells. Quantification of the ratio of TAZ/Actin signal in 5 different Western blot analyses.

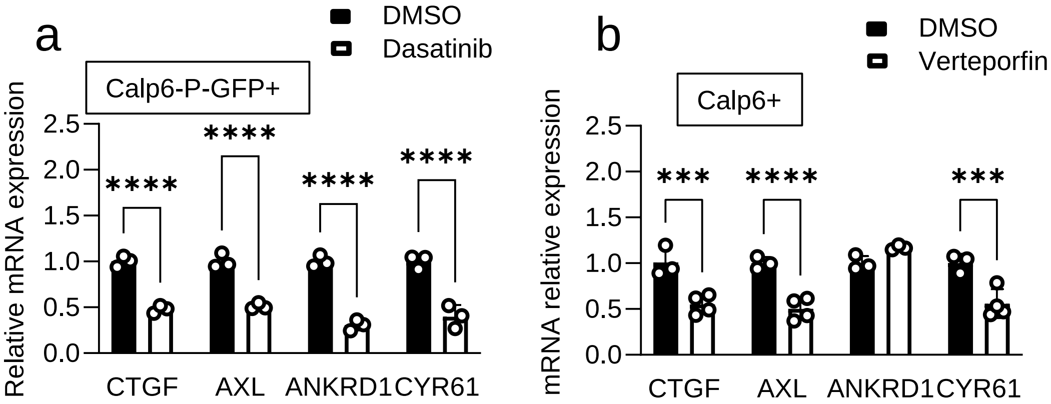

Supplementary Figure 4 : **Reactivation of the Hippo pathway and direct inhibition of YAP/TAZ activity resulted in different modulation of target genes.** Expression of YAP/TEAD targets as indicated in Calp6-P-GFP+ cells (a) or calpain-6 overexpressing cells (Calp6+, b). The cells were treated for 24 hrs. with 1µM Dasatinib (a) or Verteporfin (b) or the eluent (DMSO). Results are expressed as mean % of ACT/PPIA expression ± SEM. *** and **** indicate significant difference, p<0,001 and p<0,0001, respectively.

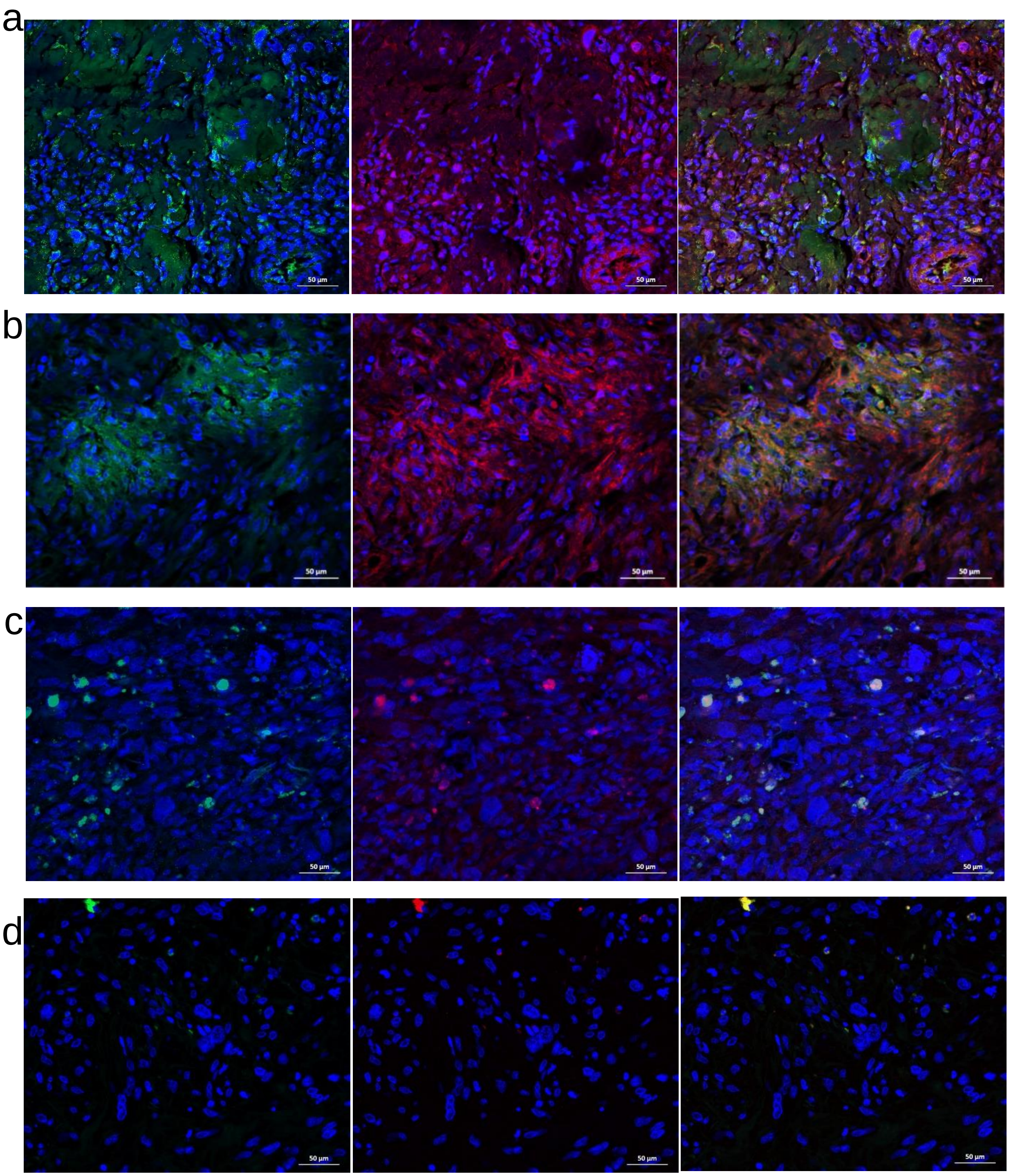

Supplementary Figure 5 **: Calpain-6 was associated with YAP expression in metastases of osteosarcoma patients**. **a**, **b**, **c**, Immunofluorescence of Calpain-6 (green) and YAP (red) in sections of paraffin-embedded lung metastasis from 3 different patients. Dapi served to stain the nucleus of cells (blue).**d**, Rabbit and mouse Ig were used to control immunolabeling of calpain-6 and YAP. White bars indicate 50 µm.

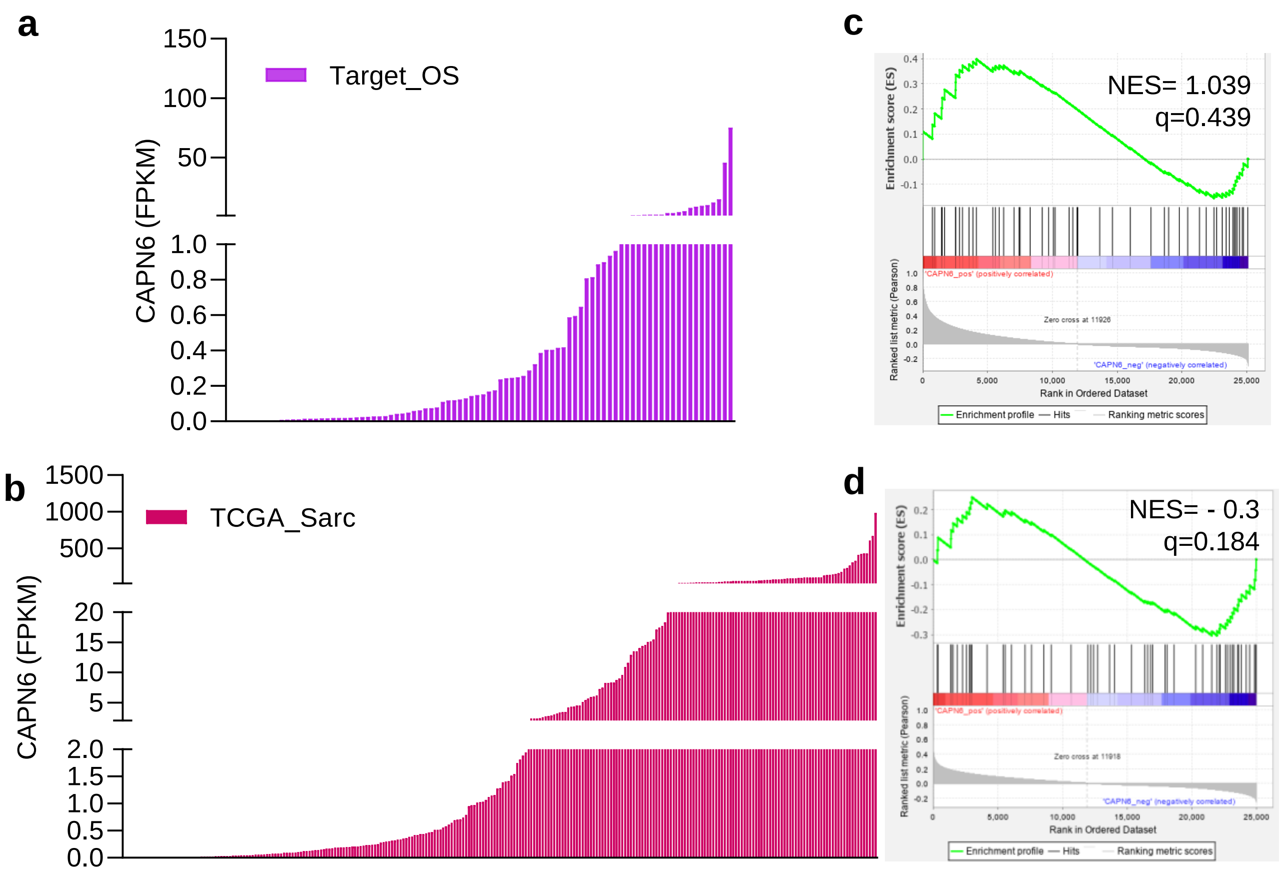

Supplementary Figure 6: **Calpain-6 was not associated with the Cordenonsi_YAP_Conserved_ Signature in sarcoma tissues. a**, **b**, Expression of calpain-6 in 88 bone (Target_OS) and 265 soft tissue (TCGA_Sarc) sarcomas. Transcriptomic data were extracted from The Cancer Genome Atlas data base. The tumors were ranked according the expression of calpain-6 gene (CAPN6) expressed as FPKM (Fragments per kilobase of exon per million reads mapped). **c**,**d**, Gene set enrichment analyses of the Cordenonsi_YAP_Conserved_Signature in bone and soft tissue sarcomas.

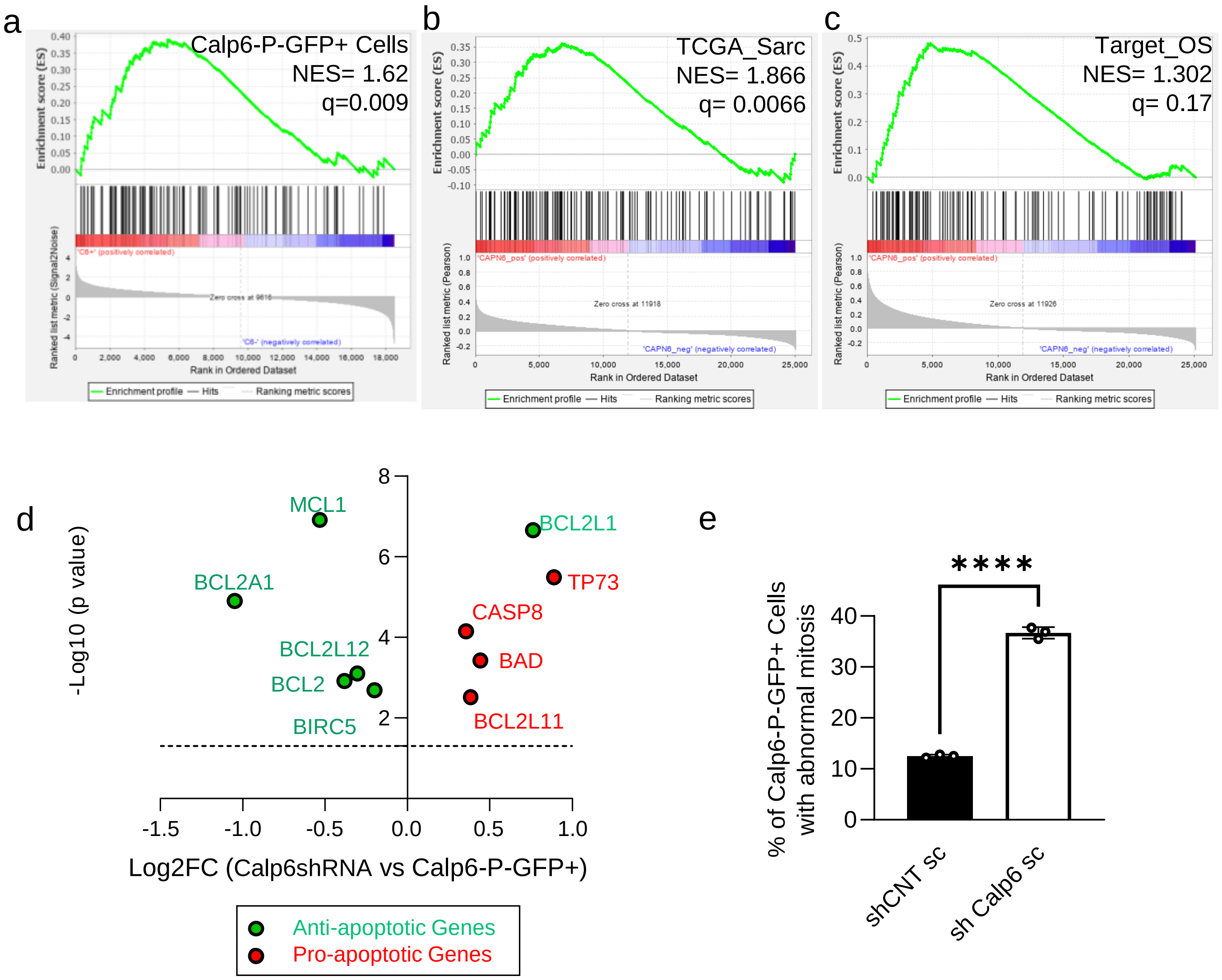

Supplementary Figure 7: **DNA repair and cell death in calpain-6-expressing osteosarcoma cells and sarcoma tissues**. **a**, **b**, **c**, Gene set enrichment analyses using the HALLMARK_DNA_REPAIR gene set in Calp6-P-GFP+ compared to Calp6 shRNA 143B cells and in bone (Target_OS) and soft tissue (TCGA_Sarc) sarcomas that were sorted according calpain-6 expression. **d**, Differential expression of genes involved in apoptosis comparing Calp6 shRNA to Calp6-P-GFP+ 143B cells. The log2 Fold Change (FC) of expression for each gene was plot against the –log10 of the p value. The dotted line indicates the threshold -Log10 (p=0,05). **e**, Effect of a different set of control and calpain-6 shRNA on mitosis in 143B Calp6-P-GFP+ 143B cells. The cells were blocked in G2 overnight. Fresh medium was then added to induce the entry into mitosis. The percentage of cells with abnormal mitosis was counted comparing control and Calp6 shRNA-expressing cells. Results are mean ± SEM of the percentage of catastrophe mitosis in 3 different cultures. **** p <0.0001

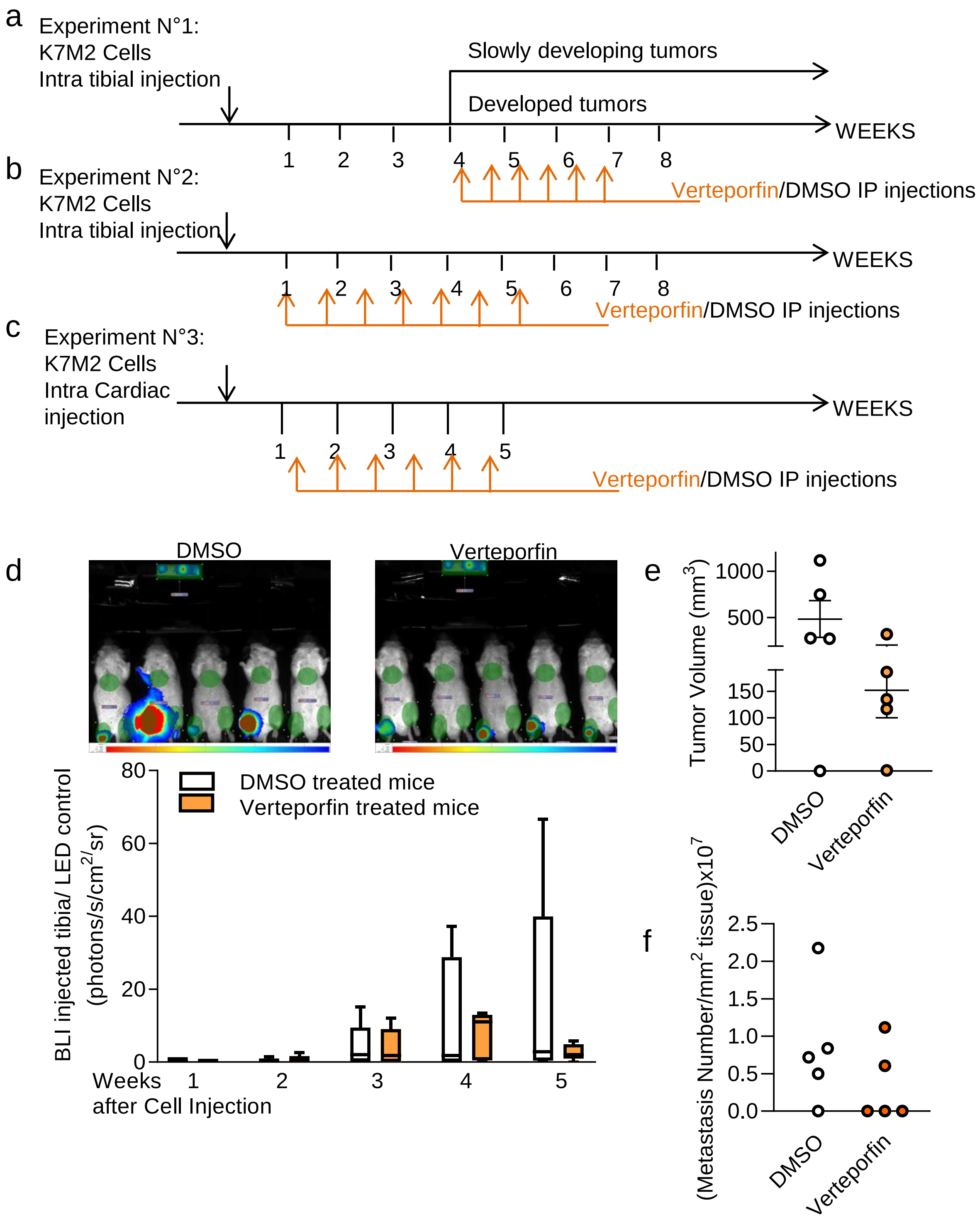

Supplementary Figure 8: **YAP inhibition using verteporfin resulted in the prevention of bone tumor out-growth**. **a**, **b**, **c**, Different experiment designs to assess the effects of verteporfin at different steps of tumor growth. **a**, K7M2 cells were implanted into the tibia of mice and the treatment (orange arrows) was started at 4 weeks. The mice were split into 2 groups according the development of the bone tumor at the onset of the treatment. **b**, K7M2 cells were implanted into the tibia of mice and the treatment (orange arrows) was started at 1 week. **c**, K7M2 cells were injected by intracardiac route and the treatment (orange arrows) was started at day 11. **d-f**, Tumor development in the 10 mice of the experience N°2. Bioluminescence detection 5 weeks after cell implantation. Quantification of the bioluminescence during tumor development. The results are expressed as bioluminescence intensity (BLI). **e**, The volume of the bone tumor 5 weeks after cell implantation. Results are mean tumor volume ± SEM of 5 mice in each group. **f**, The number of metastatic nodules in lungs 5 weeks after cell implantation .

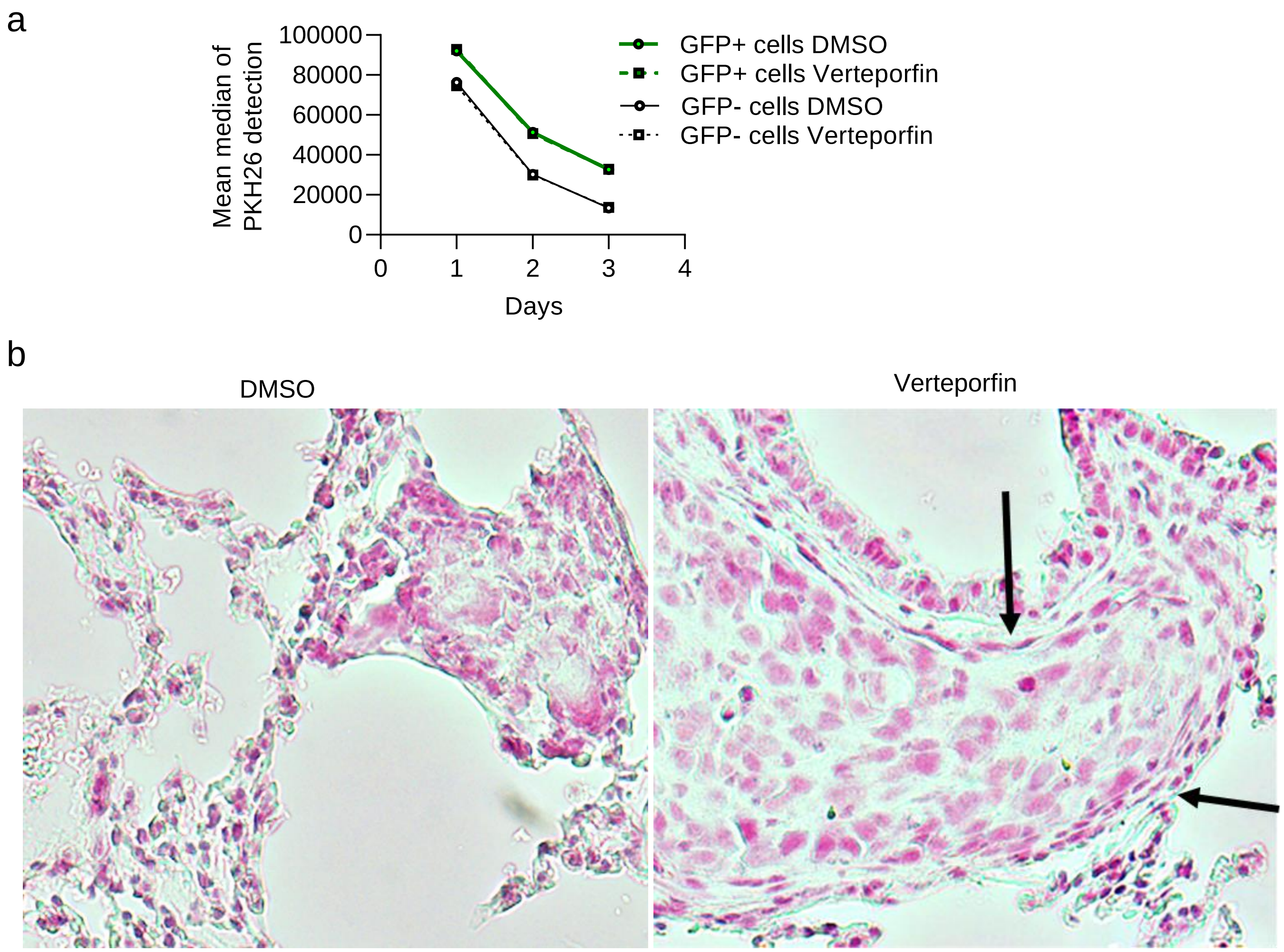

Supplementary Figure 9: **Dual effects of verteporfin on cell and tumor growth**. **a**, Effects of verteporfin on the proliferation of murine osteosarcoma cells. Membrane of Calp6-P-GFP K7M2 cells were stained with PKH26 (Day 0). The cells were cultured in the presence of 1µM verteporfin or DMSO (eluant). After 1, 2 and 3 days the cells were collected and the residual PKH26 labelling was determined by flow cytometry. The Calp6-P-GFP – and GFP+ cells were sorted for PKH26 fluorescence intensity measurement (MFI). **b**, H&E staining of sections of metastases in lungs of DMSO vs verteporfin-treated mice. The arrows show inflammatory cells around the nodules.
