# Supplementary material for: A calpain-6/YAP axis in sarcoma stem cells that drives the outgrowth of tumors and metastases
Source: Cell Death Dis. 2022 Sep 24;13(9):819. doi: 10.1038/s41419-022-05244-3 (PMC9509353; doi:10.1038/s41419-022-05244-3)

Western blot Figure 2a

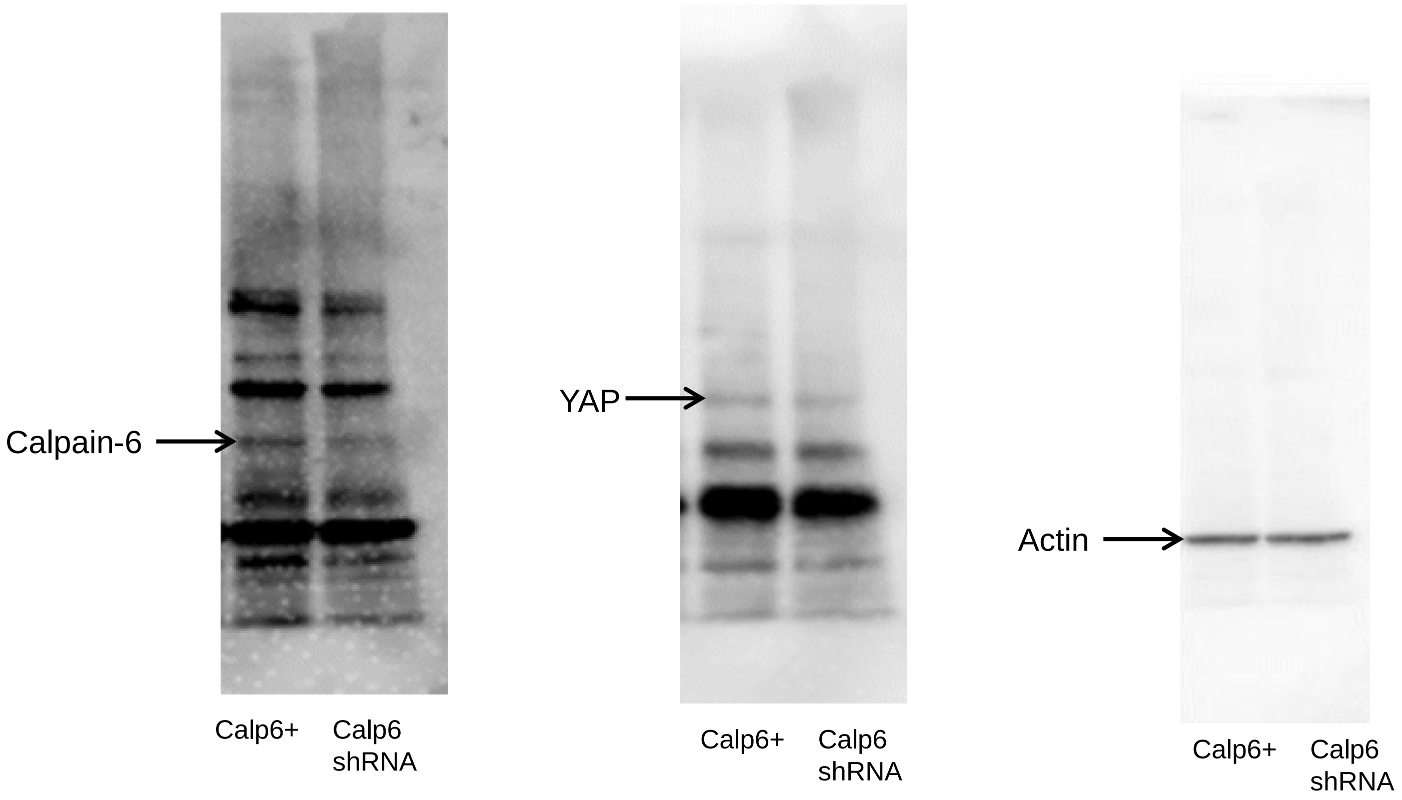

Western blot Supplementary Figure 3d

Calpain-6

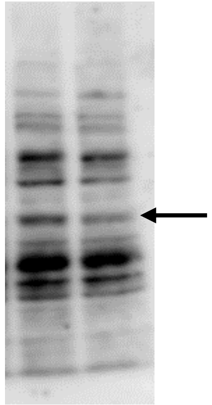

TAZ

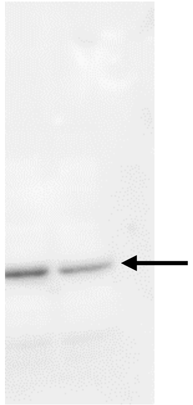

Actin

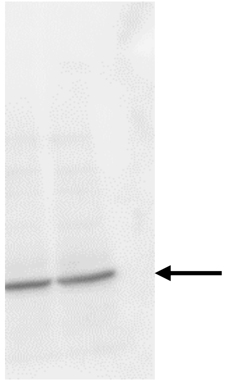

Western blot Figure 2c

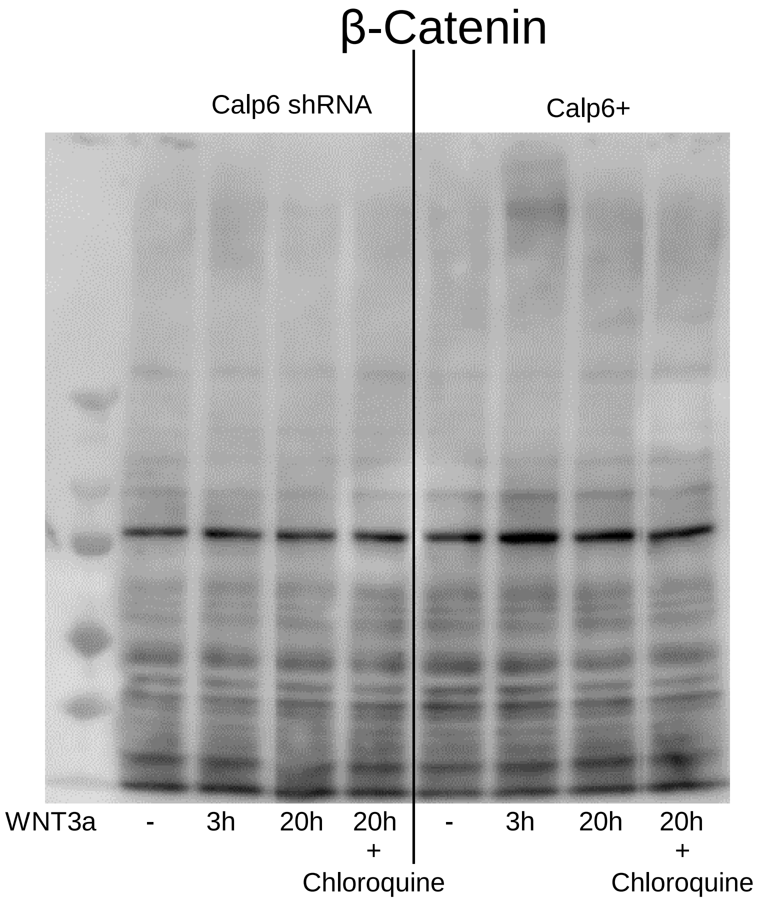

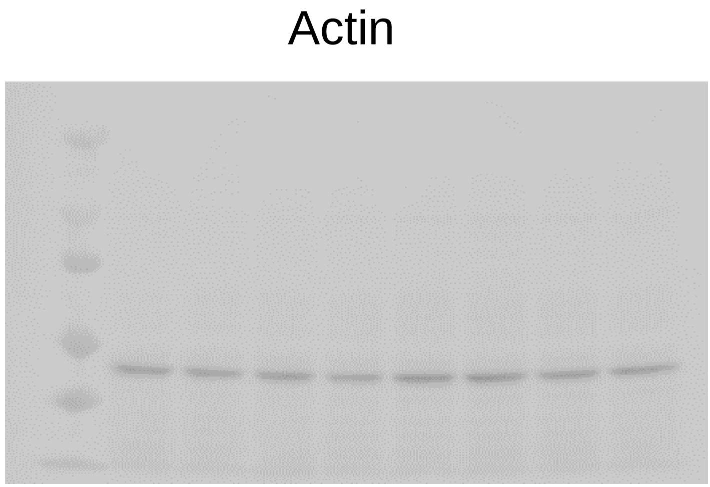

Western blot Figure 2e

Axin GSK3

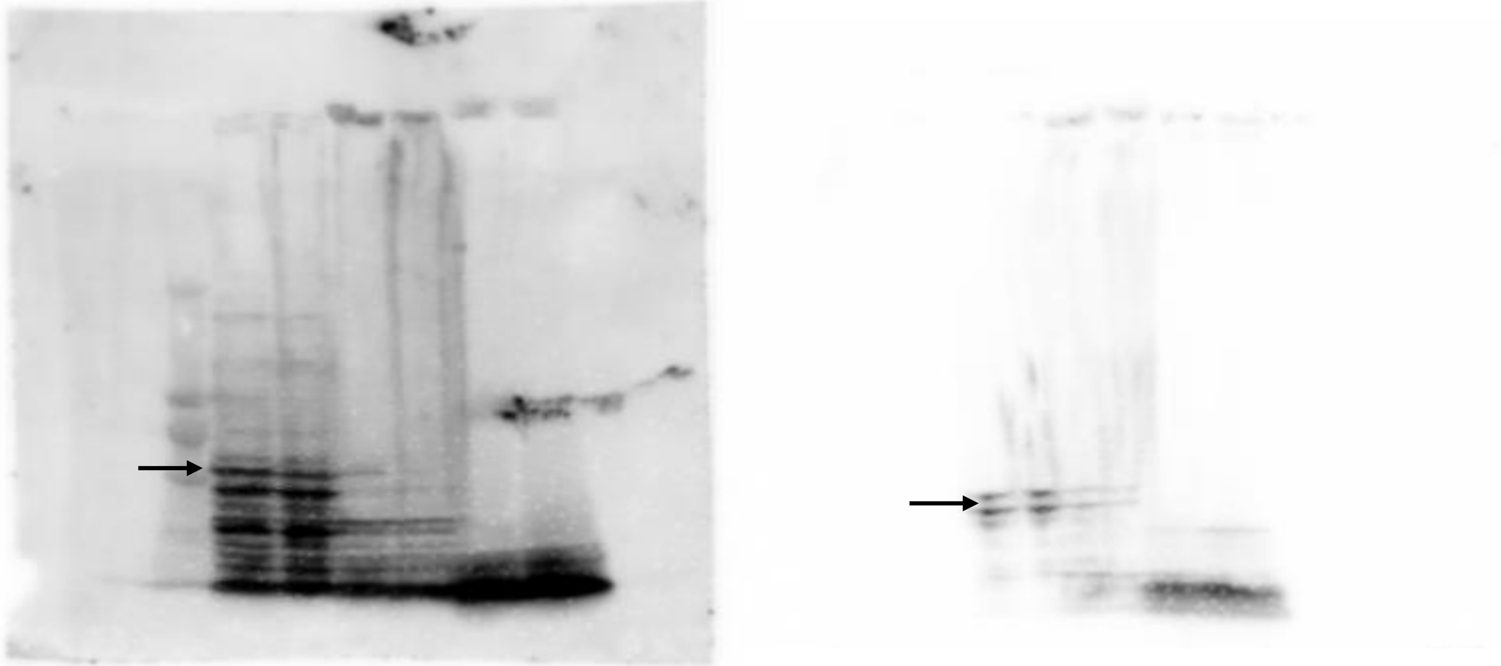

Supplement: Supplementary file 3 — Original Data File [file 41419_2022_5244_MOESM3_ESM.pdf]
